# Comparative effectiveness of oral antidiabetic drugs in preventing cardiovascular mortality and morbidity: A network meta-analysis

**DOI:** 10.1371/journal.pone.0177646

**Published:** 2017-05-25

**Authors:** Gyeongsil Lee, Seung-Won Oh, Seung-Sik Hwang, Ji Won Yoon, Sungchan Kang, Hee-Kyung Joh, Hyuktae Kwon, Jeehyun Kim, Danbee Park

**Affiliations:** 1 Department of Family Medicine, Seoul National University Hospital, Seoul, Korea; 2 Department of Family Medicine, Healthcare System Gangnam Center, Seoul National University Hospital, Seoul, Korea; 3 Department of Public Health Science, Graduate School of Public Health, Seoul National University, Seoul, Korea; 4 Department of Internal Medicine, Healthcare System Gangnam Center, Seoul National University Hospital, Seoul, Korea; 5 Department of Medicine, Seoul National University College of Medicine; Department of Family Medicine, Seoul National University Health Service Center, Seoul, Korea; Universita degli Studi di Perugia, ITALY

## Abstract

In the Guidance for Industry from the Food and Drug Administration in 2008, excess cardiovascular risk should be ruled out in trials of all new antidiabetic drugs; however, relatively few studies have focused on cardiovascular safety with antidiabetic drug use. We aimed to examine mortality and cardiovascular risk using a network meta-analysis. We searched the Medline, Embase, Cochrane, and ClinicalTrials.gov registry databases in March 2016 to identify randomized controlled trials reporting cardiovascular risk with the following oral antidiabetic drugs: metformin, sulfonylureas, thiazolidinedione (TZD), dipeptidyl peptidase-4 (DPP4) inhibitors, and sodium-glucose co-transporter-2 (SGLT2) inhibitors. We assessed the differences in the risks of all-cause mortality, cardiovascular-related mortality, acute coronary syndrome (ACS), and myocardial infarction (MI) among antidiabetic drugs with fixed effect models for direct pairwise comparisons and Bayesian network meta-analyses to integrate direct and indirect comparisons. Of the 101,183 patients in 73 randomized controlled trials, 3,434 (3.4%) died. The relative risks of all-cause mortality with SGLT2 inhibitor use were 0.68 (95% credible interval: 0.57–0.80), 0.74 (0.49–1.10), 0.63 (0.46–0.87), 0.71 (0.55–0.90), and 0.65 (0.54–0.78), compared with placebo, metformin, sulfonylurea, TZD, and DPP4 inhibitor, respectively. The relative risks of cardiovascular-related mortality with SGLT2 inhibitor use were 0.61 (0.50–0.76), 0.81(0.36–1.90), 0.52(0.31–0.88), 0.66(0.49–0.91), and 0.61(0.48–0.77), compared with placebo, metformin, sulfonylurea, TZD, and DPP4 inhibitor, respectively. The relative risks of ACS with SGLT2 inhibitor use was consistent with that of all-cause mortality. SGLT2 inhibitor use was associated with a lower risk of ACS than the other OADs and placebo. The relative risks of MI with SGLT2 inhibitor use were 0.77 (0.63–0.93) and 0.75 (0.60–0.94), compared with placebo and DPP4 inhibitor, respectively. The currently available data provide the evidence of cardiovascular benefit from use of SGLT2 inhibitors to patients with type 2 diabetes, although additional results from ongoing studies will be pivotal.

## Introduction

Diabetes, given the burden of associated morbidity and mortality [1], particularly related to cardiovascular disease (CVD), is one of the most challenging diseases globally. The risk of CVD in patients with diabetes is approximately twice that of people without diabetes [2]. CVD prevention is a main goal of diabetes treatment. Intensive glycemic control reduces the overall microvascular complication rate by 25%, compared with conventional treatment [3]; however, the effect on macrovascular complications is unclear. Further, there is concern about the cardiovascular safety of some oral antidiabetic drugs (OADs). After Nissen and Wolski reported that rosiglitazone was likely to increase the risk of myocardial infarction (MI) and cardiovascular-related mortality [4], the Food and Drug Administration issued an updated Guidance for Industry in 2008 requiring that pre- and post-approval studies for all new antidiabetic drugs rule out excess cardiovascular risk [5].

Recently developed drugs, such as dipeptidyl peptidase-4 (DPP4) and sodium-glucose co-transporter-2 (SGLT2) inhibitors, are expected to have cardiovascular benefits owing to the low risk of hypoglycemia and weight gain [6,7]. However, most trials failed to demonstrate a reduction in the risk of cardiovascular mortality or morbidity, although these drugs were deemed safe [2,8]. The most recently reported randomized controlled trial (RCT), the Empagliflozin Cardiovascular Outcome Event Trial in Type 2 Diabetes Mellitus Patients (EMPA-REG OUTCOME) trial, showed that SGLT2 inhibitor use decreases the risk of cardiovascular events, compared with placebo [9]. However, more trials are necessary to confirm the cardiovascular benefit and superiority of newly developed drugs over standard drugs.

The choice of OAD in the initial treatment of diabetes is an important issue. The American Diabetes Association and European Association for the Study of Diabetes recommend metformin as the first-line drug based on efficacy, safety, and cost [10,11]. However, other antidiabetic drugs may also be used, based on individual clinical profiles. To date, uncertainty remains regarding whether specific antidiabetic drugs have greater cardiovascular benefit over others. Therefore, we aimed to compare all-cause and cardiovascular mortality and morbidity among OADs, by conducting a network meta-analysis.

## Materials and methods

### Search strategy and selection criteria

Our systematic review protocol was drafted using guidance from the Preferred Reporting Items for Systematic reviews and Meta-Analyses Extension for Network Meta-analysis (PRISMA-NMA) [12]. We searched the MEDLINE and EMBASE, the Cochrane Central Register of Controlled Trials, and the ClinicalTrials.gov registry for articles published through March 2016 (S1 Table). The OADs targeted in our comparison were metformin, sulfonylureas, thiazolidinedione (TZD), dipeptidyl peptidase 4 (DPP4) inhibitors, sodium glucose cotransporter 2 (SGLT2) inhibitors, alpha-glucosidase inhibitors, and meglitinide. When searching the ClinicalTrials.gov registry, we did not use the outcome keywords. As a result, we reviewed all registered clinical trials including unpublished reports about OADs that met our comparison criteria. We included not only pre-specified cardiovascular outcome but also cardiovascular events reported as severe adverse events (SAEs). We supplemented information in the registry reports with that from pharmaceutical company websites for missing outcome data. Additional publications were retrieved from the bibliographies of relevant manuscripts when they were considered potentially pertinent. All citations were eligible for inclusion regardless of publication year or language.

After removing duplicate citations, two authors (GSL and SWO) independently screened the titles and abstracts to identify potentially relevant citations. Full texts were then reviewed to establish whether all pre-specified inclusion criteria were met: participants were adults with type 2 diabetes; group allocation was based on OAD use; the study reported at least two groups; participants were followed up for at least 24 weeks; more than 100 participants were randomized; and the number of all-cause and cardiovascular-related deaths, and the incidence of acute coronary syndromes (ACS) or MI were reported according to individual OADs. We excluded studies that examined dual treatment or injection drugs. Study authors were contacted by email to obtain additional details. Disagreement regarding study inclusion or exclusion was resolved by discussion with the other reviewers (SSH and JWY).

### Data extraction and risk of bias assessment

One author used a standardized form to extract data from each included study; a second verified data accuracy and completeness. The following were recorded: data sources (published articles, ClinicalTrials.gov registries, or pharmaceutical company registries); lead author and year of publication; study design and phase; period of the study; antidiabetic drug use at enrolment; participant age, percentage of male participants, race, and baseline hemoglobin (Hb) A1c level; duration of diabetes; duration of follow-up; outcomes measured; number of randomized participants who used each OAD; and number of participants who experienced each outcome or SAE.

We did not impose limitations on the background medications. If there was no change in the use of a background medication, we did not include that background medication in the analysis. For trials designed to compare adding a new drug to the background medication while increasing the dose of the specific background medication, the data were regarded as a comparison between a new drug and that background medication. When trials switched from a placebo to an active drug, we determined the group based on which treatment was administered for a longer duration. Separate drug dosages were not evaluated in the analyses. When one trial registered two NCT numbers in ClinicalTrials.gov separately due to an extended study duration, we used the results from the extended duration. We prioritized published data when both published and unpublished data were available.

Cardiovascular-related deaths consisted of fatal MI, fatal stroke, and sudden cardiac death. MI consisted of non-fatal MI reported in published articles and acute MI or MI reported as an SAE. ACS consisted of ACS, acute MI, or unstable angina from SAE data. The Cochrane Collaboration’s tool for assessing the risk of bias was used to examine the quality of eligible RCTs [13]. Both the manuscript and protocol, if available online, were reviewed for relevant information on quality. The risk of bias was assessed by one author (GSL) and cross-checked by a second author (SWO).

### Data synthesis and analysis

We undertook pairwise meta-analyses for within-study comparisons between one OAD and placebo or other OADs using Mantel-Haenszel fixed-effects models [14], and reported the results as relative risks (RRs) and corresponding 95% confidence intervals (CIs). Heterogeneity was measured with the *I*^*2*^ statistic. We then constructed a network meta-analysis to combine the direct and indirect evidence, following methods described by van Valkenhoef and colleagues to simultaneously compare cardiovascular risk among OADs using a Bayesian fixed-effects model [15]. Using a log risk ratio model with non-informative prior distributions, we applied a normal prior with a mean of 0 and large variance of 10,000 for each trial mean log ratio. A uniform prior range of 0–10 was used for the between-study variance component. We performed Markov Chain Monte Carlo simulations in JAGS 4.1.0 software (GNU General Public License) to obtain consistent and simultaneous estimates of all interventions using a *gemtc* R package. The first 5,000 iterations were discarded to minimize the bias of initial values as the chain reaches its target distribution. The subsequent 20,000 iterations were used to compute the estimates. The results are reported as posterior median RRs with corresponding 95% credible intervals (CrIs).

Model inconsistency was examined using Stata (version 14.0, StataCorp LP, College Station, TX, USA) by contrasting direct and indirect estimates in each triangular loop [16]. The proportion to the weight was checked via a contribution plot. The absence of small-study effects in the network dataset was checked through a comparison-adjusted funnel plot. We performed four sensitivity analyses to assess the robustness of the results: first, data from double-blind RCTs; second, data from published articles; third, trials with >500 patients and >1-year durations; and fourth, data from pre-specified outcome as cardiovascular morbidity or mortality. Sub-analyses were conducted, stratified by age (<65 years and ≥65 years), baseline HbA1c (<8.0 and ≥8.0), duration of diabetes diagnosis (<10 years and ≥10 years), year of study initiation (before and after 2008), and in patients with high risk (renal impairment, atherosclerosis disease, chronic heart failure, and old age).

## Role of the funding source

The funder played no role in the study design; data collection, analysis, or interpretation; or writing of the report. GSL, SWO, and SSH had full access to all study data. The corresponding author had final responsibility for the decision to submit for publication.

## Results

### Study and patient characteristics

**Fig 1:**
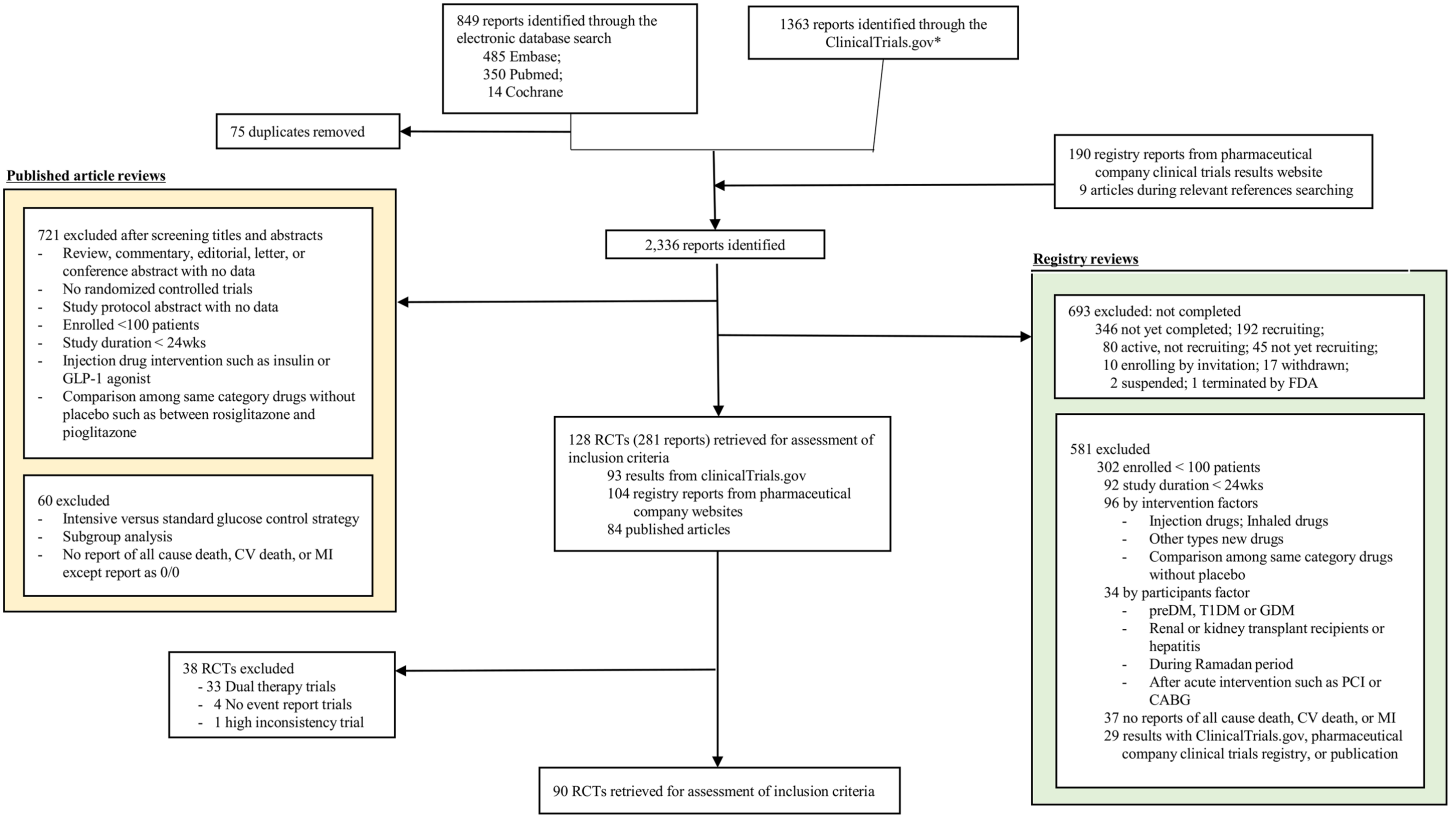
PRISMA-NMA diagram. We searched the MEDLINE and EMBASE, the Cochrane Central Register of Controlled Trials, and the ClinicalTrials.gov registry for articles published through March 2016 (S1 Table). The OADs targeted in our comparison were metformin, sulfonylureas, thiazolidinedione (TZD), dipeptidyl peptidase 4 (DPP4) inhibitors, sodium glucose cotransporter 2 (SGLT2) inhibitors, alpha-glucosidase inhibitors, and meglitinide. *When searching the ClinicalTrials.gov registry, we did not use the outcome keywords. As a result, we reviewed all registered clinical trials including unpublished reports about OADs that met our comparison criteria. We included not only pre-specified cardiovascular outcome but also cardiovascular events reported as severe adverse events (SAEs).

Fig 1 shows the flow diagram of this study according to the PRISMA-NMA statement. The literature search identified 2,336 reports. After screening the titles and abstracts, 2,055 reports did not meet the predetermined selection criteria. The full texts of the remaining 281 reports (128 RCTs) were reviewed. Dual therapy intervention trials including fixed-dose medication (n = 33) and trials reporting no outcome event (n = 4) were excluded. One acarbose trial was excluded because of high levels of inconsistency, which confidence intervals for the ratio of two odds ratios (RoR) of this trial was not compatible with zero inconsistency. Finally, 90 RCTs were included in the final analysis. S2 Table summarizes the characteristics of these 90 RCTs of sulfonylureas, metformin, TZDs, DPP4 inhibitors, and SGLT2 inhibitors.

The mean age was 54–73 years in each study. Baseline HbA1c levels were 7.2–8.9%, and the duration from diabetes diagnosis ranged from naïve to a mean of 18 years. Participants were diverse in terms of race—including white, Asian, and multi-race. The assessment of the risk of bias is presented in S3 Table and S1 Fig. Overall, 30–40% of publications provided details about randomization and allocation concealment procedures. Three trials were open-label RCTs.

### All-cause mortality

**Fig 2:**
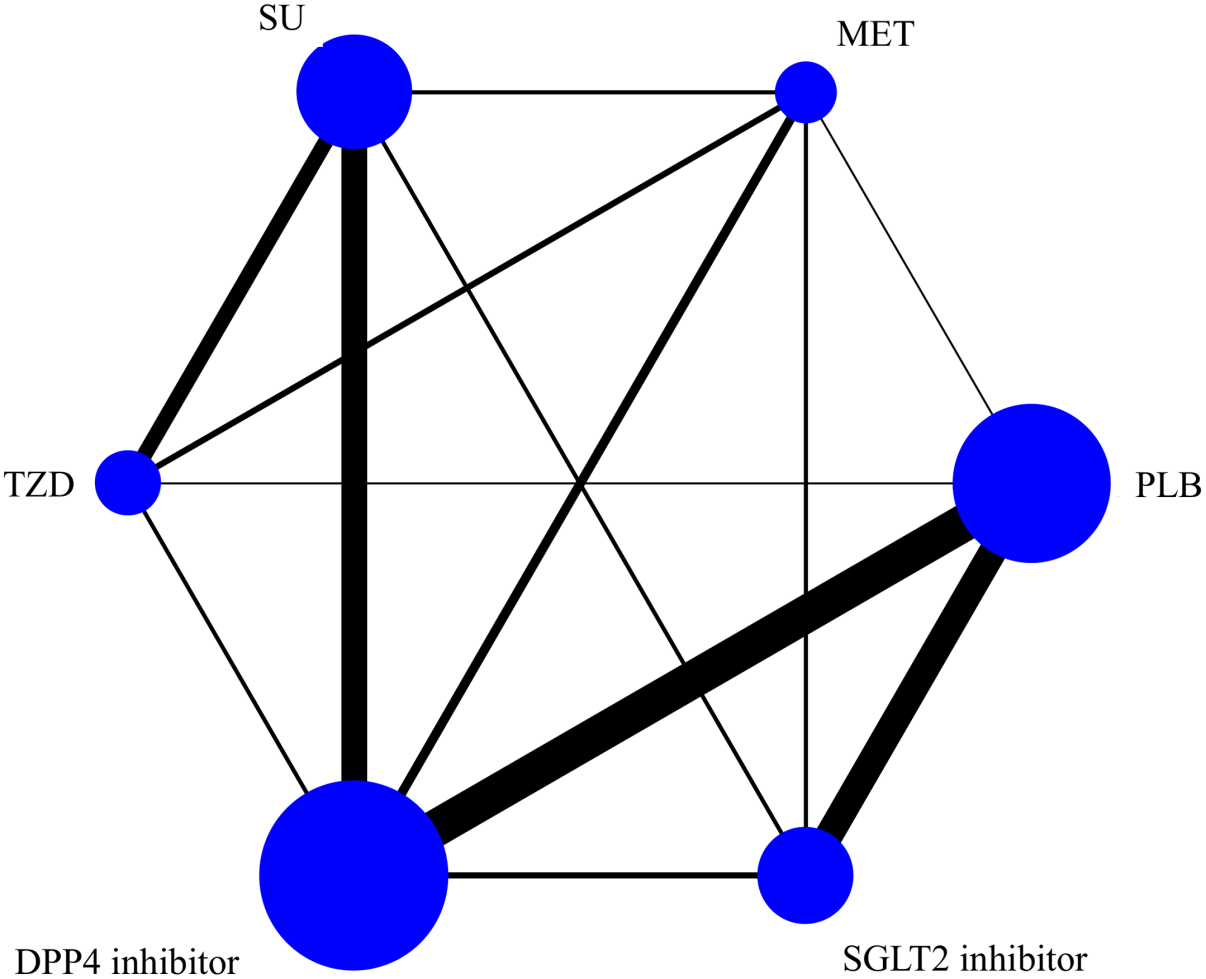
Network of oral antidiabetic drugs comparison of all-cause mortality for the network meta-analysis. Each circle node represents a drug included in the analysis and the size of circle is proportional to the number of patients randomly assigned to the drug. Each line corresponds to direct comparison between drugs and the width of line is proportional to the number of trials comparing each pair of treatments. TZD: thiazolidinedione. DPP4: dipeptidyl peptidase-4. SGLT2: sodium glucose cotransporter-2.

**Fig 3:**
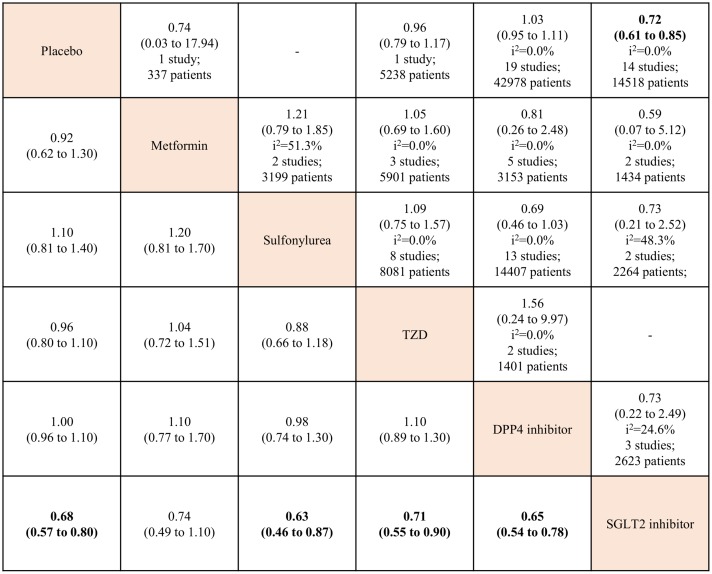
Network and pairwise meta-analyses for all-cause mortality of oral antidiabetic drugs. Traditional pairwise (upper right side) and network (lower left side) meta-analytic results are depicted for the all-cause mortality. Outcome of meta-analysis is expressed as relative risks (RRs) (95% confidence intervals) in the case of pairwise meta-analysis and (95% credible intervals) in the case of network meta-analysis. For the pairwise meta-analysis, RRs of less than 1 indicate that the drug located in the column is safer. For the network meta-analysis, RRs of less than 1 indicate that the drug located in the row is safer. Bold results indicate statistical significance. The analyses of all-cause mortality risk included data from 73 RCTs but the sum of total studies is 75 because two trials split in four. One is A Diabetes Outcome Progression Trial (ADOPT) [29], three-arm (metformin, glyburide, and rosiglitazone) study, which split in three. The other is Rosiglitazone Evaluated for Cardiovascular Outcomes and regulation of Glycaemia in Diabetes (RECORD) [31] which split in two after searching reports as separated by metformin or sulfonylurea at ClinicalTrials.gov website. TZD: thiazolidinedione. DPP4: dipeptidyl peptidase-4. SGLT2: sodium glucose cotransporter-2.

The analysis of all-cause mortality risk included data from 73 RCTs reporting 3,434 (3.4%) deaths in 101,183 patients. Fig 2 shows the network plot of eligible comparisons. The results of pairwise and network meta-analyses for all-cause mortality are summarized in Fig 3. In the pairwise meta-analysis, SGLT2 inhibitor use was associated with a lower risk of all-cause mortality compared with placebo, based on data from 14,518 patients in 14 studies (RR, 0.72; 95% CI, 0.61–0.85; *I*^*2*^ = 0.0%). There were no significant differences between other OADs and placebo. In the network meta-analysis, only SGLT2 inhibitor use was associated with reduced all-cause mortality, compared with placebo (RR, 0.68; 95% CrI, 0.57–0.80). Within the active OADs, SGLT2 inhibitor use was associated with a lower risk of mortality, compared with sulfonylureas, TZD, and DPP4 inhibitors (RR, 0.63; 95% CrI, 0.46–0.87; RR, 0.71; 95% CrI, 0.55–0.90; and RR, 0.65; 95% CrI, 0.54–0.78, respectively). There were no significant differences between the other OADs and placebo.

We examined the contribution of direct estimates to mixed and indirect estimates (S2 Fig). Regarding the loop-specific approach, the inconsistency between direct and indirect estimates of all-cause mortality was significantly low (S3 Fig). Through the comparison-adjusted funnel plot, we confirmed these data were quite symmetric, indicating rare small-study effects in the network (S4 Fig). Four sensitivity analyses (data from double-blinded RCTs, published articles, large scale trials with >500 patients and >1-year durations, and pre-specified cardiovascular outcome trials) were performed to examine the robustness of the results: these results were very similar to those of the main analysis (S5 Fig).

The results of the sub-analysis stratified by age, baseline HbA1c, duration from diabetes diagnosis, year of study initiation, and cardiovascular risk status are shown in S6 Fig. All-cause mortality in the sub-analysis of patients <65 years (62 of 73 trials) was similar to that in the full data analysis, in which SGLT2 inhibitors were associated with a lower risk than placebo, sulfonylurea, TZD, and DPP4 inhibitors. In the sub-analysis of patients ≥65 years (9 of 73 trials) that did not include any metformin and TZD trials, we found no significant differences between drugs. The sub-analysis of baseline HbA1c ≥8.0 (31 of 73 trials) showed a significant decrease in all-cause mortality associated with SGLT2 inhibitor use compared with placebo and DPP4 inhibitor use. On the other hand, the HbA1c <8 sub-analysis (31 of 73 trials) did not show any significant differences. In the sub-analysis by duration of diabetes diagnosis, both groups (<10 years and ≥10 years) showed a significant decrease in all-cause mortality associated with SGLT2 inhibitor use compared with placebo and DPP4 inhibitor use. There were no significant differences between drugs in the sub-analysis of high-risk patients, such as the elderly and patients with renal impairment, patients with cardiovascular disease, or patients at a risk of cardiovascular disease (21 of 73 trials). The sub-analysis excluding rosiglitazone was similar to the full data analysis (66 of 73 trials). In the sub-analysis of trials starting after 2008 (39 of 73 trials), during which period there were no TZD trials, SGLT2 inhibitor use was associated with reduction in all-cause mortality compared with the use of placebo and DPP4 inhibitors. Otherwise, sub-analysis of trials before 2008 (33 of 73 trials) showed no significant result.

### Cardiovascular-related mortality and morbidity

**Fig 4:**
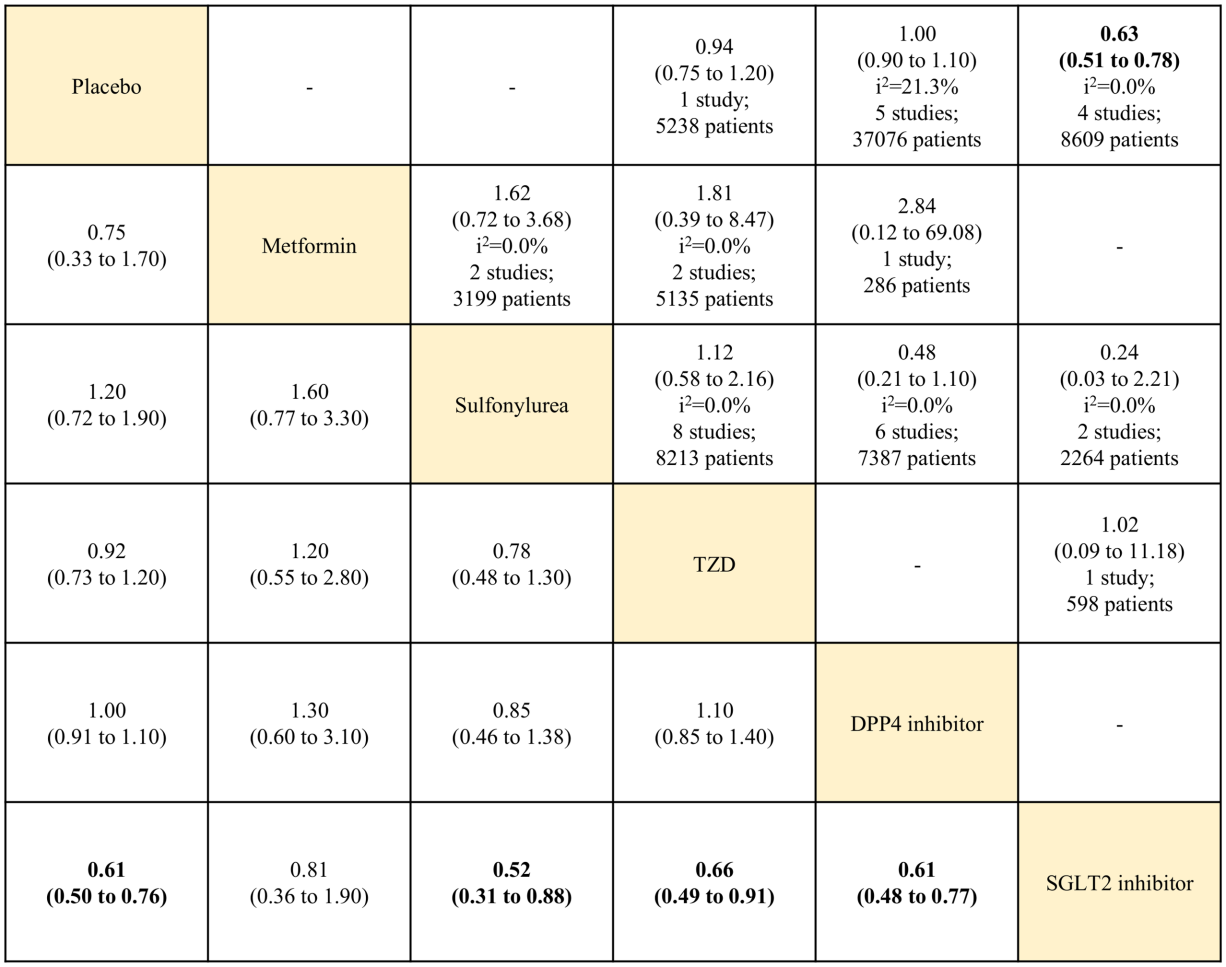
Network and pairwise meta-analyses for cardiovascular-related mortality of oral antidiabetic drugs. Traditional pairwise (upper right side) and network (lower left side) meta-analytic results are depicted for the cardiovascular-related mortality. Outcome of meta-analysis is expressed as relative risks (RRs) (95% confidence intervals) in the case of pairwise meta-analysis and (95% credible intervals) in the case of network meta-analysis. For the pairwise meta-analysis, RRs of less than 1 indicate that the drug located in the column is safer. For the network meta-analysis, RRs of less than 1 indicate that the drug located in the row is safer. Bold results indicate statistical significance. The analyses of all-cause mortality risk included data from 29 RCTs but the sum of total studies is 32 because two trials split in four. One is A Diabetes Outcome Progression Trial (ADOPT) [29], three-arm (metformin, glyburide, and rosiglitazone) study, which split in three. The other is Rosiglitazone Evaluated for Cardiovascular Outcomes and regulation of Glycaemia in Diabetes (RECORD) [31] which split in two after searching reports as separated by metformin or sulfonylurea at ClinicalTrials.gov website. TZD: thiazolidinedione. DPP4: dipeptidyl peptidase-4. SGLT2: sodium glucose cotransporter-2.

Analysis of cardiovascular-related mortality included data from 29 RCTs reporting 2,148 (2.9%) deaths in 73,189 patients. In the pairwise meta-analysis, only SGLT2 inhibitor use was associated with a lower risk of cardiovascular-related mortality, compared with placebo, for 8609 patients in four studies (RR, 0.63; 95% CI, 0.51–0.78; *I*^2^ = 0.0%). In the network meta-analysis, SGLT2 inhibitor use also reduced cardiovascular-related mortality, compared with placebo (RR, 0.61; 95% CrI, 0.50–0.76). Within the active OADs, SGLT2 inhibitor use was associated with a lower risk of cardiovascular-related mortality, compared with sulfonylureas, TZD, and DPP4 inhibitors (RR, 0.52; 95% CrI, 0.31–0.88; RR, 0.66; 95% CrI, 0.49–0.91; and RR, 0.61; 95% CrI, 0.48–0.77, respectively) (Fig 4).

**Fig 5:**
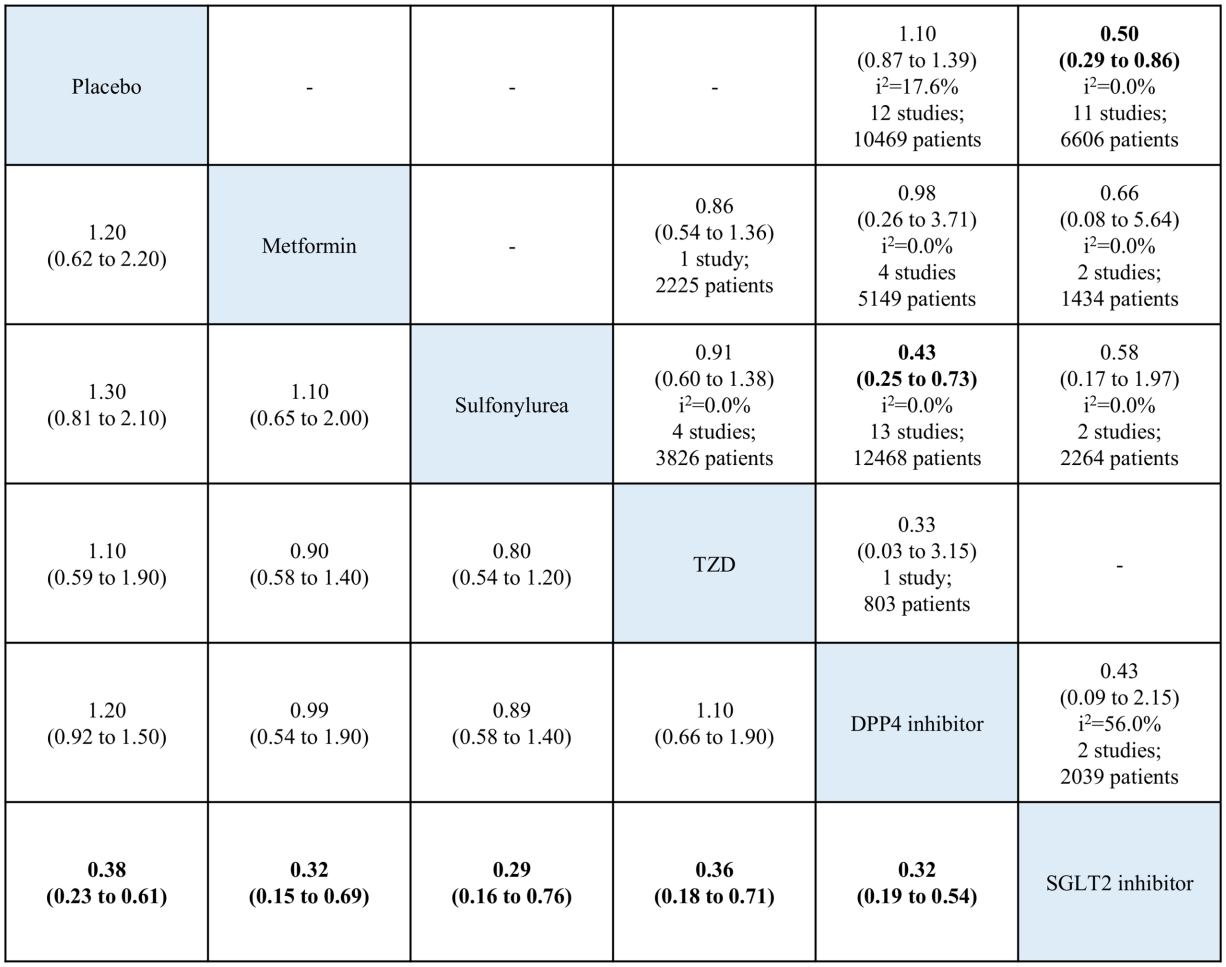
Network and pairwise meta-analyses for acute coronary syndrome of oral antidiabetic drugs. The analyses of acute coronary syndrome risk included data from acute coronary syndrome, acute myocardial infarction or unstable angina counted as severe adverse events. Traditional pairwise (upper right side) and network (lower left side) meta-analytic results are depicted for the cardiovascular-related mortality. Outcome of meta-analysis is expressed as relative risks (RRs) (95% confidence intervals) in the case of pairwise meta-analysis and (95% credible intervals) in the case of network meta-analysis. For the pairwise meta-analysis, RRs of less than 1 indicate that the drug located in the column is safer. For the network meta-analysis, RRs of less than 1 indicate that the drug located in the row is safer. Bold results indicate statistical significance. The analyses included data from 51 RCTs but the sum of total studies is 52 because two trials split in two. Rosiglitazone Evaluated for Cardiovascular Outcomes and regulation of Glycaemia in Diabetes (RECORD) [31] split in two after searching reports as separated by metformin or sulfonylurea at ClinicalTrials.gov website. TZD: thiazolidinedione. DPP4: dipeptidyl peptidase-4. SGLT2: sodium glucose cotransporter-2.

The ACS risk analysis included data from 51 RCTs. In the pairwise meta-analysis including data for 6606 patients in 11 studies, SGLT2 inhibitor use was associated with decreased ACS risk, compared with placebo (RR, 0.50; 95% CI, 0.29–0.86; I^2^ = 0.0%). There were no significant differences between the other OADs and placebo. Within the active OADs, based on data for 12468 patients in 13 studies, DPP4 inhibitor use was associated with decreased ACS risk, compared with sulfonylureas (RR, 0.43; 95% CI, 0.25–0.73; I^2^ = 0.0%). In the network meta-analysis, SGLT2 inhibitor use was associated with a lower risk of ACS than the other OADs, including placebo (Fig 5).

**Fig 6:**
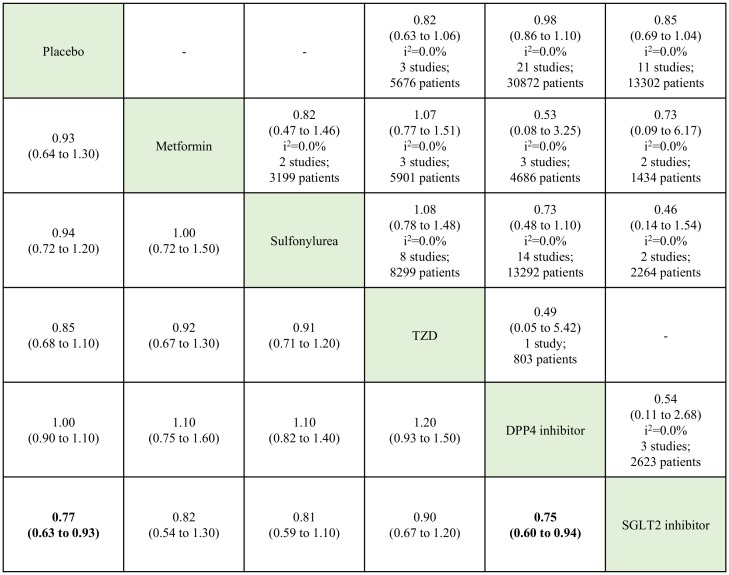
Network and pairwise meta-analyses for myocardial infarction of oral antidiabetic drugs. The analyses of myocardial infarction risk included data from not only published article as non-fatal myocardial infarction but unpublished reports as acute myocardial infarction or myocardial infarction as severe adverse events. Traditional pairwise (upper right side) and network (lower left side) meta-analytic results are depicted for the cardiovascular-related mortality. Outcome of meta-analysis is expressed as relative risks (RRs) (95% confidence intervals) in the case of pairwise meta-analysis and (95% credible intervals) in the case of network meta-analysis. For the pairwise meta-analysis, RRs of less than 1 indicate that the drug located in the column is safer. For the network meta-analysis, RRs of less than 1 indicate that the drug located in the row is safer. Bold results indicate statistical significance. The analyses of all-cause mortality risk included data from 70 RCTs but the sum of total studies is 73 because two trials split in four. One is A Diabetes Outcome Progression Trial (ADOPT) [29], three-arm (metformin, glyburide, and rosiglitazone) study, which split in three. The other is Rosiglitazone Evaluated for Cardiovascular Outcomes and regulation of Glycaemia in Diabetes (RECORD) [31] which split in two after searching reports as separated by metformin or sulfonylurea at ClinicalTrials.gov website. TZD: thiazolidinedione. DPP4: dipeptidyl peptidase-4. SGLT2: sodium glucose cotransporter-2.

The analyses of MI risk included data from 70 RCTs (Fig 6). The network meta-analysis showed a lower risk of MI associated with SGLT2 inhibitor use than placebo (RR, 0.77; 95% CrI, 0.63–0.93) or DPP4 inhibitors (RR, 0.75; 95% CrI, 0.60–0.94). S7, S8 and S9 Figs depict the network-, contribution-, inconsistency-, and comparison-adjusted funnel- plots for cardiovascular-related mortality, ACS, and MI, respectively. The inconsistency of direct with indirect estimates of cardiovascular-related mortality, ACS, and MI were significantly low based on the loop-specific approach. The comparison-adjusted funnel plot of cardiovascular-related mortality, ACS, and MI depicted were quite symmetric, meaning rare small-study effects in the network.

## Discussion

In this network meta-analysis, we identified 90 RCTs that reported the risk of all-cause mortality or adverse cardiovascular outcomes for OADs. SGLT2 inhibitor use provided significant benefits for all-cause and cardiovascular-related mortality and morbidity in patients with type 2 diabetes, compared with both placebo and other OADs such as sulfonylureas, TZD, and DPP4 inhibitors.

Our findings are consistent with a prior conventional meta-analysis of 57 trials of seven different SGLT2 inhibitors compared with placebo, that reported lower RRs for all-cause and cardiovascular-related mortality [17]. The authors explained that these results were affected by one large-scale trial, EMPA-REG OUTCOME, which showed lower all-cause and cardiovascular-related mortality in the empagliflozin group compared with the placebo group [9]. While there were no significant differences in the risk of MI between SGLT2 inhibitor and placebo use in EMPA-REG OUTCOME, SGLT2 inhibitor use was associated with a decreased risk of MI compared with placebo in the present study. Moreover, SGLT2 inhibitors also decreased ACS risk in our study, even though EMPA-REG OUTCOME was not included in our analysis of ACS risk. Therefore, other SGLT2 inhibitors as well as empagliflozin could decrease cardiovascular complications. Further studies are necessary to demonstrate the beneficial effect of other SGLT2 inhibitors.

SGLT2 inhibitors also had a favorable effect on all-cause and cardiovascular mortality compared with sulfonylureas, TZD, and DPP4 inhibitors in our network meta-analysis. This finding is plausible considering the mechanisms and effects of each drug. SGLT2-mediated glucose reabsorption, which drives excess sodium reabsorption, might cause volume expansion, and SGLT2 inhibitors would block this effect [18]. Reduced renal glucose and sodium reabsorption—therefore lower blood glucose and cardiac preload—would lead to reduced body weight and systolic blood pressure, a potentially powerful protective mechanism against heart failure. Sulfonylureas act on pancreatic beta-cells, independently of serum glucose levels, to enhance insulin secretion, potentially causing adverse hypoglycemic events. This can prolong the QT interval and is associated with cardiac ischemia [19], increasing the risk of arrhythmias, acute MI, and sudden cardiac death [8]. A previous meta-analysis also reported a lower risk of hypoglycemia with SGLT2 inhibitors than with sulfonylureas [20]. In addition, weight gain caused by sulfonylureas or TZD could exacerbate cardiovascular risks [2,8].

A recently reported network meta-analysis by Palmer and colleagues showed different results from our analysis in that there were no significant differences in the risk of cardiovascular or all-cause mortality between the antidiabetic drug classes [21]. The difference arises from two points. First, their analysis included monotherapy trials and dual or triple therapy trials added to metformin or metformin + sulfonylurea, which were baseline medications. However, our analysis included a greater variety of studies in terms of background medications, if there was no change in the use of them. For example, in EMPA-REG OUTCOME, background medications were to remain unchanged after randomization. Therefore, it was included in our analysis, but was excluded in that of Palmer and colleagues. Second, there was a difference in the statistical methodology used. Our analysis was performed in a Bayesian setting, whereas Palmer and colleagues’ analysis was based on a frequentist setting. Bayesian estimation is usually used in network meta-analysis to avoid the frequentist assumption of a normal approximation of the estimated study-specific treatment effects. Frequentist estimation could be problematic in small count data [22]. In Palmer and colleagues’ report, 25 studies were included in the analysis for cardiovascular death and they had few or no events [21]. Twenty-nine studies were included in our analysis for cardiovascular mortality, with a small number of events. Additionally, a considerable number of loops included values of high inconsistency in Palmer and colleagues’ analysis for cardiovascular mortality. The maximum RoR and 95% CI for cardiovascular mortality data were 12.35 and 1.00–556.17, respectively [21]. Inconsistency between direct and indirect comparison is an important issue in network meta-analysis; when the RoR score is too high, inconsistency cannot be excluded [23].

Cardiovascular risk differs according to patient characteristics. Older patients, those with suboptimal glycemic control, and those with a longer duration from diabetes diagnosis, are at higher risk of cardiovascular events [2,24]. Hence, we performed a subgroup analysis stratified by age, cardiovascular-risk status, baseline HbA1c, and duration from diabetes diagnosis. However, the findings were not significantly different to those of the full data analysis. In addition, the Guidance for Industry 2008 update requires that excess cardiovascular risk associated with new antidiabetic drugs be ruled out [5]. We thus performed a sub-analysis according to year of study. In this sub-analysis, trials published after 2008 did not include TZD, and the superiority of SGLT2 inhibitors was maintained.

Metformin, the first-line medication for treating type 2 diabetes, provided cardiovascular protection in previous studies [10,11]. However, there was no difference in all-cause or cardiovascular mortality and MI risk between metformin and placebo or other OADs in our study. We did not include early large trials such as the UK Prospective Diabetes Study (UKPDS) [3] or Action to Control Cardiovascular Risk in Diabetes (ACCORD) [25] which focused on intensive versus standard glycemic control instead of a head-to-head comparison between OADs. In most recent RCTs focused on the efficacy and safety of individual newly developed drugs, metformin was usually included in the baseline medications. Therefore, few metformin trials were included in our analysis. Only two RCTs (NCT00528372 and NCT01368081) that directly compared metformin and SGLT2 inhibitors were included: the pairwise meta-analysis revealed no significant difference. Events occurred in one of 274 patients and two of 1160 patients, respectively [26,27]. Hence, the association between metformin use and higher ACS risk compared with SGLT2 inhibitors in our network meta-analysis should be interpreted with caution.

There was no difference between metformin and sulfonylurea in all-cause or cardiovascular mortality, different to results reported in a recent systematic review. Maruthur and colleagues concluded that metformin is associated with a lower risk of cardiovascular mortality than sulfonylureas [28], based on the individual results of two RCTs (A Diabetes Outcome Progression Trial [ADOPT] and Study on the Prognosis and Effect of Antidiabetic Drugs on Type 2 Diabetes Mellitus with Coronary Artery Disease [SPREAD-DIMCAD]) [29,30] and several observational studies. In our study, the direct comparison between metformin and sulfonylurea was based on the same two RCTs. However, the results were not significant in either the pairwise or the network meta-analysis.

There were also no significant differences between TZD and placebo or other OADs, except SGLT2 inhibitors, similar to recent studies. In the RECORD trial (the basis of the withdrawal of the previous restriction of rosiglitazone), overall cardiovascular morbidity and mortality in the rosiglitazone group were not different from those in the metformin and sulfonylurea groups [31]. Several studies of pioglitazone reported that it did not increase the risk of cardiovascular complications [32–35]. In the PROspective pioglitAzone Clinical Trial In macrovascular Events (PROactive) clinical trial, pioglitazone reduced the composite of all-cause mortality, non-fatal MI, and stroke in high-risk patients with type 2 diabetes [32]. The potential discrepancy in cardiovascular risk between rosiglitazone and pioglitazone might stem from their distinct effects on the lipid profile. In a meta-analysis, pioglitazone decreased triglycerides and increased high-density lipoprotein cholesterol [36]. Therefore, we performed a sub-analysis excluding rosiglitazone data, but could not demonstrate a difference.

DPP4 inhibitors decrease serum glucose levels with fewer hypoglycemia events and less weight gain [37], which may be cardio-protective. However, the comparison between DPP4 inhibitors and placebo in the present study did not show any difference in all-cause and cardiovascular mortality, consistent with previous RCTs and meta-analyses. Three prior large RCTs suggested that DPP4 inhibitors do not improve cardiovascular endpoints compared with placebo, except for the risk of hospitalization for heart failure [38–40]. However, DPP4 inhibitors were associated with a reduction in ACS compared with sulfonylureas in our pairwise meta-analysis. This result is plausible considering the aforementioned differing mechanisms of action and side effects of the two drugs.

Our study has certain limitations. First, we aimed to compare the difference between the classes of OADs, not individual drugs. However, individual drugs within the same class could differ. Onset time and affected organs could differ among sulfonylureas [41,42]. In a recent network meta-analysis, gliclazide and glimepiride were associated with a lower risk of all-cause and cardiovascular mortality, compared with glibenclamide [43]. Second, we did not distinguish between doses of individual drugs owing to high levels of inconsistency when the analysis was stratified by dosage. With additional studies a more robust conclusion could be reached. Third, we did not consider rescue or background medications owing to their diverse use. Additionally, we could not consider the influence of other drugs like antihypertensive drugs or statins because most RCTs have not provided information on these drugs. Thus, the effect of add-on medication, or of drug-to-drug interactions, was not evaluated. Fourth, we did not consider causality between OADs and individual death events because many trials did not report that.

Our analyses utilized a systematic and comprehensive approach. The data quality in almost all included RCTs (including unpublished reports) was relatively high. Robustness was confirmed via sensitivity and subgroup analyses. Furthermore, we compared the effect of each OAD class with placebo and with other OAD classes through conventional and network meta-analyses. The currently available data provide evidence of a cardiovascular benefit with SGLT2 inhibitor use in patients with type 2 diabetes. However, additional results from ongoing studies are pivotal.

## Supporting information

S1 PRISMA Checklist PRISMA NMA checklist of items to include when reporting a systematic review involving a network meta-analysis. (PDF)

S1 Table Search strategy on Medline, Embase, Cochrane Central Register of Controlled Trials (CENTRAL), and ClinicalTrials.gov. (PDF)

S2 Table Main characteristics of included trials. (PDF)

S3 Table Risk of bias assessment. (PDF)

S1 Fig Risk of bias assessment (summary graph). (PDF)

S2 Fig Contribution plot for network meta-analysis for all-cause mortality of oral antidiabetic drugs. (PDF)

S3 Fig Inconsistency plot for network meta-analysis for all-cause mortality of oral antidiabetic drugs assuming loop-specific heterogeneity estimates. (PDF)

S4 Fig Comparison-adjusted funnel plot for network meta-analysis of all-cause mortality on oral antidiabetic drugs. (PDF)

S5 Fig Network plots and predictive interval plot for sensitivity analysis. (PDF)

S6 Fig Network plots and predictive interval plot for sub-analysis. (PDF)

S7 Fig Network meta-analysis for cardiovascular-related mortality. (PDF)

S8 Fig Network meta-analysis for acute coronary syndrome. (PDF)

S9 Fig Network meta-analysis for myocardial infarction. (PDF)
