# Supplementary material for: Comparative effectiveness of oral antidiabetic drugs in preventing cardiovascular mortality and morbidity: A network meta-analysis
Source: PLoS One. 2017 May 25;12(5):e0177646. doi: 10.1371/journal.pone.0177646 (PMC5444626; doi:10.1371/journal.pone.0177646)
Supplement: S1 Table — (PDF) [file pone.0177646.s002.pdf]

S1 Table. Search Strategy on Medline, Embase, Cochrane Central Register of Controlled Trials (CENTRAL), and ClinicalTrials.gov

**Medline**	
**#1**	Search type 2 diabetes mellitus[MeSH Terms]
**#2**	Search (biguanide) OR metformin
**#3**	Search ((((((((((sulfonylurea) OR acetohexamide) OR carbutamide) OR chlorpropamide) OR glibenclamide) OR gliclazide) OR glimepiride) OR glipizide) OR gliquidone) OR glyburide) OR tolazamide) OR tolbutamide
**#4**	Search (((thiazolidinediones) OR glitazones) OR pioglitazone) OR rosiglitazone
**#5**	Search ((alpha glucosidase inhibitor) OR acarbose) OR miglitol
**#6**	Search (((((dipeptidyl peptidase 4 inhibitors) OR sitagliptin) OR vildagliptin) OR saxagliptin) OR linagliptin) OR gemigliptin
**#7**	Search (((meglitinide) OR repaglinide) OR nateglinide) OR mitiglinide
**#8**	Search (((sodium glucose cotransporter 2 inhibitor) OR dapagliflozin) OR canagliflozin) OR empagliflozin
**#9**	#2 OR #3 OR #4 OR #5 OR #6 OR #7 OR #8
**#10**	Search ((((((cardiovascular diseases[MeSH Terms]) OR myocardial ischemia[MeSH Terms]) OR acute coronary syndrome[MeSH Terms]) OR angina pectoris[MeSH Terms]) OR coronary disease[MeSH Terms]) OR myocardial infarction[MeSH Terms]) OR mortality[MeSH Terms]
**#11**	Search randomized controlled trial[Publication Type]
**#12**	#1 AND #9 AND #10 AND #11 Filters: Adult: 19+ years

**Embase**	
**#1**	'diabetes mellitus'/exp OR 'diabetes mellitus' OR 'type 2 diabetes'/exp OR 'type 2 diabetes'
**#2**	'biguanide' OR 'metformin'
**#3**	'sulfonylurea' OR 'acetohexamide' OR 'carbutamide' OR 'chlorpropamide' OR 'glibenclamide' OR 'gliclazide' OR 'glimepiride' OR 'glipizide' OR 'gliquidone' OR 'glyburide' OR 'tolazamide' OR 'tolbutamide'
**#4**	'thiazolidinediones' OR 'glitazone' OR 'pioglitazone' OR 'rosiglitazone'
**#5**	'alpha glucosidase inhibitor' OR 'acarbose' OR 'miglitol'
**#6**	'dipeptidyl peptidase 4 inhibitor' OR 'sitagliptin' OR 'vildagliptin' OR 'saxagliptin' OR 'linagliptin' OR 'gemigliptin'
**#7**	'meglitinide' OR 'repaglinide' OR 'nateglinide' OR 'mitiglinide'
**#8**	'sodium glucose co transporter 2 inhibitor' OR 'dapagliflozin' OR 'canagliflozin' OR 'empagliflozin'
**#9**	#2 OR #3 OR #4 OR #5 OR #6 OR #7 OR #8
**#10**	'cardiovascular disease' OR 'myocardial ischemia' OR 'acute coronary syndrome' OR 'angina pectoris' OR 'coronary disease' OR 'myocardial infarction' OR 'mortality'
**#11**	'randomized controlled trial'
**#12**	#1 AND #9 AND #10 AND #11 AND [adult]/lim AND [aged]/lim

CENTRAL	
#1	type 2 diabetes:ti,ab,kw or "diabetes mellitus":ti,ab,kw (Word variations have been searched)
#2	metformin:ti,ab,kw or "biguanide":ti,ab,kw (Word variations have been searched)
#3	sulfonylurea:ti,ab,kw or "acetohexamide":ti,ab,kw or "carbutamide":ti,ab,kw or "chlorpropamide":ti,ab,kw or "glibenclamide":ti,ab,kw (Word variations have been searched)
#4	Gliclazide:ti,ab,kw or "glimepiride":ti,ab,kw or "glipizide":ti,ab,kw or "gliquidone":ti,ab,kw or "glyburide":ti,ab,kw (Word variations have been searched)
#5	tolazamide:ti,ab,kw or "tolbutamide":ti,ab,kw (Word variations have been searched)
#6	thiazolidinedione:ti,ab,kw or "glitazone":ti,ab,kw or "pioglitazone":ti,ab,kw or "rosiglitazone":ti,ab,kw (Word variations have been searched)
#7	alpha glucosidase:ti,ab,kw or "acarbose":ti,ab,kw or "miglitol":ti,ab,kw (Word variations have been searched)
#8	dipeptidyl peptidase IV:ti,ab,kw or "sitagliptin":ti,ab,kw or vildagliptin:ti,ab,kw or saxagliptin:ti,ab,kw (Word variations have been searched)
#9	linagliptin:ti,ab,kw or gemigliptin:ti,ab,kw (Word variations have been searched)
#10	meglitinide:ti,ab,kw or "repaglinide":ti,ab,kw or "nateglinide":ti,ab,kw or "mitiglinide":ti,ab,kw (Word variations have been searched)
#11	sodium glucose co-transporter 2:ti,ab,kw or dapagliflozin:ti,ab,kw or canagliflozin:ti,ab,kw or empagliflozin:ti,ab,kw (Word variations have been searched)
#12	#2 or #3 or #4 or #5 or #6 or #7 or #8 or #9 or #10 or #11
#13	cardiovascular disease:ti,ab,kw or "myocardial infarction":ti,ab,kw or "myocardial ischemia":ti,ab,kw or "acute coronary syndrome":ti,ab,kw or "angina pectoris":ti,ab,kw (Word variations have been searched)
#14	coronary disease:ti,ab,kw or "mortality":ti,ab,kw (Word variations have been searched)
#15	#13 or #14
#16	randomized control trial:ti,ab,kw (Word variations have been searched)
#17	#1 and #12 and #15 and #16

ClinicalTrials.gov
(diabetes mellitus OR type 2 diabetes) AND (biguanide OR metformin OR sulfonylurea OR acetohexamide OR carbutamide OR chlorpropamide OR glibenclamide OR gliclazide OR glimepiride OR glipizide OR gliquidone OR glyburide OR tolazamide OR tolbutamide OR thiazolidinedione OR glitazones OR pioglitazone OR rosiglitazone OR alpha glucosidase inhibitor OR acarbose OR miglitol OR dipeptidyl peptidase 4 inhibitor OR sitagliptin OR vildagliptin OR saxagliptin OR linagliptin OR gemigliptin OR meglitinide OR repaglinide OR nateglinide OR mitiglinide OR Sodium glucose co-transporter 2 inhibitor OR dapagliflozin OR canagliflozin OR empagliflozin) AND INFLECT EXACT ("Adult" OR "Senior") [AGE-GROUP] | Completed | Exclude Unknown | Adult, Senior | Phase 2, 3, 4
