# Supplementary material for: Comparative effectiveness of oral antidiabetic drugs in preventing cardiovascular mortality and morbidity: A network meta-analysis
Source: PLoS One. 2017 May 25;12(5):e0177646. doi: 10.1371/journal.pone.0177646 (PMC5444626; doi:10.1371/journal.pone.0177646)
Supplement: S2 Table — (PDF) [file pone.0177646.s003.pdf]

S2 Table. Main Characteristics of Included Trials

NCT Number and Acronym	Publication	Trial phase	Study period (wks)	Number of randomized patients	Target	Comparator	Background medication	Population	Race or Ethnicity	Age, yr(SD)	Male sex, n(%)	Baseline HbA1c, %(SD)	Mean duration from diabetes diagnosis, yrs (SD) or [range]
**NCT00086515**	Charbonnel et al, 2006	III	104	701	SITA 100 mg qd	PLB->GLIP 5mg qd	MET	Multicentres in US	64W:14H	55 (10)	400 (57)	8.0 (0.8)	6.2 (NA)
**NCT00094770**	Seck et al, 2010	III	104	1172	SITA 100 mg qd	GLIP up to 20 mg qd	MET	Melticentres in Puerto Rico and US	D: 77C<br>S: 79C	D: 58 (9)<br>S: 57 (9)	D: 142 (57)<br>S: 161 (63)	D: 7.3 (0.6)<br>S: 7.3 (0.7)	D: 5.8 (5.7)<br>S: 5.7 (4.9)
**NCT00099866**	Schweizer et al, 2007	III	52	780	VILD 50 mg bid	MET 2000 mg qd		183 centres in 10 countries	D: 68W:20H<br>M: 70W:22H	D: 53 (12)<br>M: 54 (11)	D: 278 (53)<br>M: 146 (57)	D: 8.7 (1.1)<br>M: 8.7 (1.1)	D: 2.4 (3.4)<br>M: 2.2 (2.9)
**NCT00099905**	Dejager et al, 2007	III	24	639	VILD 50 mg qd, 50 mg bid, or 100 mg qd	PLB		134 centres in 3 countries	D50q: 73C:14H<br>D50b: 73C:13H<br>D100: 76C:15H<br>P: 69C:12H	D50q: 55 (11)<br>D50b: 53 (10)<br>D100: 54 (11)<br>P: 52 (11)	D50q: 43 (41)<br>D50b: 42 (47)<br>D100: 49 (53)<br>P: 45 (48)	D50q: 8.2 (0.8)<br>D50b: 8.6 (0.8)<br>D100: 8.4 (0.8)<br>P: 8.4 (0.8)	D50q: 2.1 (3.6)<br>D50b: 2.1 (3.3)<br>D100: 2.4 (4.2)<br>PLB: 1.6 (2.5)
**NCT00102388**	Foley et al, 2009	III	104	1092	VILD 50 mg qd	GLC 320 mg qd		151 centres in 16 countries	D: 74C:15H<br>S: 73C:15H	D: 55 (11)<br>S: 54 (10)	D: 321 (59)<br>S: 288 (53)	D: 8.6 (1.0)<br>S: 8.7 (1.1)	D: 2.4 (4.3)<br>S: 1.9 (3.1)
**NCT00106340**		III	104	3118	VILD 50 mg bid	GLM 1-6 mg qd	MET	402 centres in 25 countries	87C:8H	58 (9)	1667 (53)	7.3 (0.7)	5.7 (5.1)
**NCT00106704**	Hermansen et al, 2007	III	24	441	SITA 100 mg qd	PLB	SU or SU+MET	Multicentres in 22 countries	D: 61C:18H<br>P: 64C:15H	57 (10)	117 (53)	8.3 (0.8)	D: 8.3 (5.5)<br>P: 9.3 (6.8)
**NCT00116831 APPROACH**	Gerstein et al 2010	III	72	672	RSG 4 mg qd	GLIP 5 mg qd		92 centres in 19 countries	74W:23A	61 (9)	454 (68)	7.2 (0.9)	4.7 (0 to 35.82)
**NCT00121641**	Rosenstock et al, 2013	III	154	401	SAXA 2.5, 5, 10 mg qd	PLB		multicentres in 6 countries	85W	53 (11)	204 (51)	NA	NA
**NCT00121667**	Rosenstock et al, 2013	III	216	743	SAXA 2.5, 5, or 10 mg qd	PLB	MET	Multicentres in 9 countries	82W	55 (10)	377 (51)	8.1 (0.1)	NA
**NCT00138567**		III	52	463	VILD 50 mg bid	MET 1000 mg bid		123 centres in 7 countries	D: 72C:7B<br>M: 77C:4B	D: 54 (11)<br>M: 54 (10)	D: 167 (55)<br>M: 96 (61)	D: 8.5 (1.0)<br>M: 8.8 (1.1)	D: 2.5 (3.6)<br>M: 2.2 (2.9)
**NCT00138619**	Rosenstock et al, 2009	III	104	478	VILD 50 mg bid	RSG 8 mg qd		155 centres in 4 regions	D: 79C<br>T: 79C	D: 54 (12)<br>T: 54 (11)	D: 228 (58)<br>T: 110 (55)	D: 8.6 (1.1)<br>T: 8.6 (1.2)	D: 2.0 (2.9)<br>T: 2.6 (4.2)
**NCT00169832 VICTORY**		III	54	193	RSG	PLB	various	9 centres in Canada and Spain	NA	T: 64 (7)<br>P: 65 (7)	T: 90 (92)<br>P: 87 (92)	T: 6.9 (1.3)<br>P: 6.9 (0.8)	T; 7.8 (6.4)<br>P: 8.4 (6.9)
**NCT00174993 PROactive**	Dormandy et al, 2005	III	155	5238	PIO 15-45 mg qd	PLB	various	321 centres in 19 European countries	T: 98W<br>P: 99W	T: 62 (8)<br>P: 62 (8)	T: 1735 (67)<br>P: 1728 (66)	T: 7.8 [7.0-8.9]<br>P: 7.9 [7.1-8.9]	T: 8 [4-13]<br>P: 8 [4-14]
**NCT00225264 CHICAGO**	Mazzone et al, 2006	III	72	462	PIO 15-45 mg qd	GLM up to 4mg qd	MET or insulin	28 centres in US	T: 60W:30B<br>S: 65W:27B	T: 59 (8)<br>S: 60 (8)	T: 146 (64)<br>S: 143 (63)	7.4 (1.0)	T: 8.0 (7.6)<br>S: 7.5 (6.8)
**NCT00225277 PERISCOPE**	Nissen et al, 2008	III	72	547	PIO 15-45 mg qd	GLM 1-4 mg qd		97 centers in North America and South America,	82W:10B	60 (9)	183 (68)	7.4 (1.0)	1.4 (NA)

**NCT00279045 ADOPT**		III	4yr	4426	RSG 4 mg bid	MET 1000 mg bid; GLB 7.5 mg bid		488 centres in 17 countries	T: 87W<br>M: 89W<br>S: 89W	T: 56 (10)<br>M: 58 (10)<br>S: 56 (10)	T: 811 (57)<br>M: 864 (59)<br>S: 836 (58)	7.4 (0.9)	<2yr (96%)
**NCT00286442**	Nauck et al, 2009	III	26	527	ALO 12.5, 25 mg qd	PLB	MET	115 centres in 15 countries	P: 76W<br>D1: 80W<br>D2: 76W	P: 56 (11)<br>D1: 55 (11)<br>D2: 54 (11)	P: 50 (48)<br>D1: 101 (47)<br>D2: 114 (54)	P: 8.0 (0.9)<br>D1: 7.9 (0.7)<br>D2: 7.9 (0.8)	P: 6 (5)<br>D1: 6 (5)<br>D2: 6 (4)
**NCT00286468**	Pratley et al, 2009, Diabetes, Obesity and Metabolism	III	26	500	ALO 12.5, 25 mg qd	PLB	GLY	124 centres in 16 countries	71W:12A	57 (11)	261 (52)	8.1 (NA)	7.7 (5.8)
**NCT00286494**	Pratley et al, 2009, CMRO	III	26	493	ALO 12.5, 25 mg qd	PLB	PIO	125 centres in 8 countries	74W:11A	55 (10)	287 (58)	8.0 (0.8)	7.6 (5.7)
**NCT00295633**	Hollander et al, 2009	III	24	565	SAXA 2.5, 5 mg qd	PLB	TZD		D2: 56W:34A<br>D5: 53W:36A<br>P: 55W:34A	D2: 55 (10)<br>D5: 53 (11)<br>P: 54 (10)	D2: 106 (54)<br>D5: 89 (48)<br>P: 85 (46)	D2: 8.3 (1.1)<br>D5: 8.4 (1.1)<br>P: 8.2 (1.1)	D2: 5.3 (4.6)<br>D5: 5.2 (5.6)<br>P: 5.1 (5.4)
**NCT00313313**	Chacra et al, 2010	III	24	768	SAXA 2.5, 5 mg qd	GLB 2.5 mg qd add	GLB	Multicenters in 13 countries	D2: 60W:17A<br>D5: 60W1:18A<br>S: 60W:19A	D2: 55 (10)<br>D5: 55 (10)<br>S: 55 (11)	D2: 113 (46)<br>D5: 110 (44)<br>S: 123 (46)	D2: 8.4 (0.9)<br>D5: 8.5 (0.9)<br>S: 8.4 (0.9)	D2: 7.1 (5.9)<br>D5: 6.8 (5.8)<br>S: 6.8 (5.7)
**NCT00316082**		III	24	365	SAXA 2.5, 5 mg qd	PLB		Multicenters in 4 countries	NA	55 (10)	168 (46)	NA	NA
**NCT00333723**	Campbell et al, 2004	IV	24	245	RSG 8mg qd	PLB	GLY	39 centres in US	T: 66H:51B<br>P: 72H:44B	T: 52 (12)<br>P: 53 (10)	T: (53) of 117<br>P: (56) of 116	T: 9.2 (1.3)<br>P: 9.4 (1.4)	T-H: 6.5 (6.3)<br>T-B: 5.3 (3.3)<br>P-H: 6.2 (5.7)<br>P-B: 6.1 (4.6)
**NCT00350779**	Dobs et al, 2013	III	54	262	SITA 100mg qd	PLB	MET + RSG	140 centres in 18 countries	51W:31A	55 (9)	151 (58)	8.8 (1.0)	4.1 (4.4)
**NCT00359112**	Hamann et al, 2008	IV	52	544	RSG up to 8 mg qd	GLB up to 15 mg qd or GLC up to 320 mg qd	MET	118 centres in 11 countries	T: 94W<br>S: 95W	T: 59 (10)<br>S: 59 (9)	T: 155 (53)<br>S: 158 (52)	T: 8.0 (0.9)<br>S: 8.0 (1.0)	NA
**NCT00379769 RECORD**	Home et al, 2009	III	270	4447	RSG 4-8 mg qd	MET up to 2550 mg, SU	MET or SU	364 centres in 25 countries in Europe and Australasia	T//M: 99W<br>S//M: 98W<br>T//S: 99W<br>M//S: 99W	T//M: 57 (8)<br>S//M: 57 (8)<br>T//S: 60 (8)<br>M//S: 60 (8)	T//M: 601 (54)<br>S//M: 584 (53)<br>T//S: 541 (49)<br>M//S: 568 (51)	T//M: 7.8 (0.7)<br>S//M: 7.8 (0.7)<br>T//S: 8.0 (0.7)<br>M//S: 8.0 (0.7)	T//M: 6.1 (4.2)<br>S//M: 6.3 (4.4)<br>T//S: 7.9 (5.5)<br>M//S: 7.9 (5.2)
**NCT00395343**	Vilsboll et al, 2010	III	24	641	SITA 100 mg qd	PLB	INS or MET	100 ventres in 26 countries	70W	58 (9)	326 (51)	8.7 (0.9)	D: 13 (7)<br>P: 12 (6)
**NCT00432276**	Bosi et al, 2011	III	52	803	ALO 25 mg qd	PIO up to 45 mg qd	MET + PIO	Multicentres in US	62W:20A	55 (10)	414 (52)	8.2 (0.8)	7.2 (4.9)
**NCT00449930**	Aschner et al, 2010	III	24	1050	SITA 100 mg qd	MET 500mg qd - 1000 mg bid		113 centres in 23 countries	75W:12A	56 (11)	484 (46)	7.3 (0.7)	2.4 (3.7)
**NCT00500955**		III	32	389	RSG 4mg qd	SU(GLY or GLC) 5 mg	MET	88 centres in 11 countries	T: 78W<br>S: 76W	T: 60 (10)<br>S: 59 (10)	T: 123 (63)<br>S: 124 (69)	T: 8.5 (1.7)<br>S: 8.3 (1.6)	T: 8.0 (6.6)<br>S: 7.6 (5.7)
**NCT00501020**		IV	24	766	RSG 4 mg bid	MET up to 1000 mg bid	MET	63 centres in US	72C:13H	56 (11)	362 (51)	8.0 (1.2)	NA

**NCT00509236**	Arjona Ferreira et al, 2013, Am J Kidney Dis	III	54	129	SITA 25 mg qd	GLIP up to 20 mg qd		31 centres in 12 countries	D: 44A:25W<br>S: 49A:28W	D: 61 (9)<br>S: 59 (10)	D: 40 (63)<br>S: 37 (57)	D: 7.9 (0.7)<br>S: 7.8 (0.7)	D: 19 [12-24]<br>S: 16 [11-23]
**NCT00509262**	Arjona Ferreira et al, 2013, Diabetes Care	III	54	426	SITA 25 or 50 mg qd	GLIP up to 20 mg qd		Multicentres in 25 countries	D: 53A:30W<br>S: 59A:28W	64 (10)	253 (60)	7.8 (0.7)	D; 10.7 (7.5)<br>S: 10.1 (7.8)
**NCT00513630 SPREAD-DIMCAD**	Hong et al, 2013	III	162	304	GLP up to 30 mg daily	MET up to 1500 mg daily		15 centres in China	100A	S: 64 (9)<br>M: 63 (9)	S: 114 (77)<br>M: 122 (78)	7.6 (1.7)	S: 5.6 (4.9)<br>M: 5.6 (5.3)
**NCT00521742**	Giles et al, 2010	III	52	300	PIO 15, 30 mg qd	GLB 2.5, 5 mg qd	MET	66 centres in Mexico and US	61C	64 (NA)	168 (56)	8.6 (1.5)	T: 7.7 (NA)<br>S: 7.2 (NA)
**NCT00521820**	Giles et al, 2008	III	24	518	PIO 30 mg qd	GLB 10 mg qd		NA	T: 69C<br>S: 66C	T: 64 (10)<br>S: 63 (9)	T: 184 (70)<br>S: 197 (77)	T: 8.7 (1.6)<br>S: 9.0 (1.8)	T: 11.8 (9.2)<br>S: 11.7 (9.5)
**NCT00528372**	Bailey et al, 2015	III	102	274	DAPA 2.5, 5, or 10mg qd	PLB->MET 500mg qd		85 centres in 4 countries	95W	52 (10)	276 (49)	7.9 (0.9)	2.0 (2.9)
**NCT00528879**	Bailey et al, 2010, 2013	III	102	546	DAPA 2.5, 5, or 10mg qd	PLB	MET	80 centres in 5 countries	88W	54 (10)	292 (53)	8.0 (2.4)	6.1 (5.6)
**NCT00575588**	Goke et al, 2011	III	104	891	SAXA 5 mg qd	GLIP 2.5-20 mg qd	MET	130 centres in 11 countries	D: 82W<br>S: 84W	58 (10)	D: 212 (50)<br>S: 232 (54)	D: 7.7 (NA)<br>S: 7.7 (NA)	D: 5.5 [0-32]<br>S: 5.4 [0-34]
**NCT00601250**		III	24	701	LINA 5 mg qd	PLB	MET	82 centres in 10 countries	76W	57 (10)	379 (54)	8,1 (0.9)	NA
**NCT00602472**	Owns et al, 2011	III	24	1058	LINA 5 mg qd	PLB	SU + MET	100 centres in 11 countries	52A:47W	58 (10)	498 (47)	8.1 (0.8)	≤1:1-5:>=5(%)<br>D: 7:26:67<br>P: 4:25:71
**NCT00614939**	Nowicki et al, 2011	III	52	170	SAXA 2.5 mg qd	PLB		multicentres in 14 countries	100W	67 (9)	73 (43)	8.3 (1.2)	16.7 (8.0)
**NCT00622284**	Gallwitz et al, 2012	III	104	1552	LINA 5 mg qd	GLM up to 4 mg qd	MET	209 centres in 16 countries	85W:12A	60 (9)	933 (60)	7.7 (0.9)	≤1:>1&≤5:>5<br>D: 7:41:52<br>S: 8:39:54
**NCT00641043**		III	24	389	LINA 5 mg qd	PLB	PIO	43 centres in 7 countries	75W:25A	58 (10)	237 (61)	8.6 (0.8)	NA
**NCT00646542**	Lukashevich et al, 2011	III	24	515	VILD 50 mg qd	PLB	various	95 centres in 13 countries	ModD: 70E:15A<br>ModP: 73E:12A<br>SevD: 49E:29H<br>SevP: 50E:27H	ModD: 68 (9)<br>ModP: 70 (7)<br>SevD: 64 (9)<br>SevP: 65 (11)	ModD: 96 (58)<br>ModP: 80 (62)<br>SevD: 65 (52)<br>SevP: 53 (55)	ModD: 7.8 (1.0)<br>ModP: 7.8 (0.9)<br>SevD: 7.7 (1.0)<br>SevP: 7.7 (1.0)	ModD: 15.0 (9.1)<br>ModP: 15.2 (10.0)<br>SevD: 17.3 (8.6)<br>SevP: 19.0 (9.6)
**NCT00660907**	Nauck et al, 2011	III	52	1217	DAPA 10 mg qd	GLIP 20 mg qd	MET	95 centres in 10 countries	81W:8A	58 (10)	441 (55)	7.7 (0.9)	NA
**NCT00663260**	Kohan et al, 2014	II/III	104	252	DAPA 5, 10 mg qd	PLB	various	111 centers in 13 countries	SG5: 78W<br>SG10: 91W<br>P: 82W	SG5: 66 (8.9)<br>SG10: 68 (7.7)<br>P: 67 (8.6)	SG5: 55 (66)<br>SG10: 56 (66)<br>P: 53 (63)	SG5: 8.3 (1.0)<br>SG10: 8.2 (1.0)<br>P: 8.5 (1.3)	SG5: 16.9 (9.0)<br>SG10: 18.2 (10.1)<br>P: 15.7 (9.5)
**NCT00673231**	Wilding et al, 2014	III	104	800	DAPA 2.5, 5/10, 10 mg qd	PLB	various	126 centres in 13 countries	P: 96W<br>SG2.5: 94W<br>SG5: 95W<br>SG10: 95W	P: 59 (9)<br>SG2.5: 60 (8)<br>SG5: 60 (8)<br>SG10: 59 (9)	P: 95 (49)<br>SG2.5: 100 (50)<br>SG5: 100 (47)<br>SG10: 87 (45)	P: 8.5 (0.8)<br>SG2.5: 8.5 (0.8)<br>SG5: 8.6 (0.9)<br>SG10: 8.6 (0.8)	P: 13.5 (7.3)<br>SG2.5: 13.6 (6.6)<br>SG5: 13.1 (7.8)<br>SG10: 14.2 (7.3)

**NCT00680745**	Strojek et al, 2011	III	24	597	DAPA 2.5, 5, or 10mg qd	PLB	GLM	84 centres in 7 countries	69W:31A	60 (10)	285 (48)	8.1 (0.7)	7.5 (5.7)
**NCT00698932**		III	24	568	SAXA 5 mg qd	PLB		Multicentres in 4 countries	NA	51 (10)	315 (55)	8.1 (0.1)	NA
**NCT00701090**	Arechavaleta et al, 2011	III	30	1035	SITA 100 mg qd	GLM 1-6 mg qd	MET	Multicenters in 22 countries	D: 58W:21A<br>S: 57W:21A	D: 56 (10)<br>S: 56 (10)	D: 284 (55)<br>S: 279 (54)	D: 7.5 (0.7)<br>S: 7.5 (0.8)	D: 6.8 (4.6)<br>S: 6.7 (4.8)
**NCT00707993**		III	52	441	ALO 25 mg qd	GLP 5 mg qd	various	110 centres in 11 countries	73W:10A	70 (4)	198 (45)	NA	6.1 (6.3)
**NCT00740051**		III	52	227	LINA 5 mg qd	PLB->GLM		53 centers in 7 countries	NA	57 (10)	88 (39)	8.1 (0.9)	NA
**NCT00765830**		III	28	349	VILD 50mg qd	PLB	various	86 centres in 13 countries	Mod: 69C:16H<br>Severe: 51C:29H	Mod: 68 (8)<br>Severe: 64 (10)	Mod: 125 (59)<br>Severe: 82 (52)	NA	NA
**NCT00790205 TECOS**	Bethel et al, 2015, Green et al, 2015	III	162	14735	SITA 100 mg qd	PLB	various	673 sites in 38 countries	68W:15A	66 (8)	10416 (71)	7.3 (0.7)	Median [IQR] : 9.4 [4.9, 15.3]
**NCT00855166**	Bolinder et al, 2014	III	102	182	DAPA 10 mg qd	PLB	MET	multicentres in 5 countries	100W	61 (8)	100 (56)	7.2 (0.5)	5.8 (NA)
**NCT00856284 ENDURE**	Prato et al, 2014	III	104	2639	ALO 12.5, 25 mg qd	GLIP 5mg qd	MET	multicentres in 32 countries	62W:23A:8B	55 (10)	1312 (50)	7.6 (0.6)	NA
**NCT00885352**	Fonseca et al, 2013	III	26	313	SITA 100 mg qd	PLB	MET + PIO	58 centres in 12 countries	D: 48C:21A:14B<br>P: 53C:22A:12B	56 (9)	195 (62)	8.7 (1.0)	D: 9.4 (5.8)<br>P: 10.2 (6.1)
**NCT00894868**		III	52	798	VILD 50 mg bid	PLB	various	67 centres in 15 countries	65C:24A	63 (9)	195 (77)	7.8 (1.0)	9.3 (7.9)
**NCT00968708 EXAMINE**	White et al, 2013	III	160	5380	ALO 25, 12.5, 6.25 mg qd	PLB	various	898 centres in 49 countries	73W:20A	61 [NA]	D: 1828 (68)<br>P: 1823 (68)	8.0 (1.1)	D: 7.1 [2.6-13.8]<br>P: 7.3 [2.8-13.7]
**NCT00968812 CANTATA-SU**	Cefalu et al, 2013	III	104	1452	CANA 100, 300 mg qd	GLM	MET	157 centres in 19 countries	67W:20A	56 (9)	756 (52)	7.8 (0.8)	6.6 (5.3)
**NCT00984867**	Jabbour et al, 2014	III	24	451	DAPA 10 mg qd	PLB	SITA or SITA + MET	Multicentres in 6 countriess	D: 72W<br>P: 76W	55 (10)	245 (55)	7.9 (0.8)	5.7 (NA)
**NCT00996658**	Bajaj et al, 2014	III	24	278	LINA 5 mg qd	PLB	MET + PIO	Multicenters in 4 countries	NA	54 (9)	132 (49)	L: 8.4 (0.1)<br>P: 8.5 (0.1)	NA
**NCT01006590 PROMPT**	Hermans et al, 2012	III/IV	24	286	SAXA 5 mg qd	MET up to 1500 mg qd	MET	7 European countries	D: 99W<br>M: 97W	D: 59 (11)<br>M: 59 (10)	D: 88 (60)<br>M: 76 (55)	D: 7.7 (0.9)<br>M: 7.8 (0.8)	D: 6.0 (5.3)<br>M: 6.9 (6.0)
**NCT01006603 GENERATION**	Schernthaner et al, 2015	III/IV	52	720	SAXA 5 mg qd	GLM 1-6 mg qd	MET	152 centres in 12 European countries and Mexico	D: 98W<br>S: 99W	D: 73 (6)<br>S: 73 (5)	D: 217 (60)<br>S: 228 (63)	D: 7.6 (0.7)<br>S: 7.6 (0.7)	D: 7.6 (6.4)<br>S: 7.6 (6.0)
**NCT01011868**		II	78	494	EMPA 10, 25 mg qd	PLB	INS ± MET ± SU	Multicentres in 7 countries	67W	59 (10)	276 (56)	NA	NA
**NCT01031680**	Cefalu et al, 2015	III	52	922	DAPA 10 mg qd	PLB	various	Multicentres in 9 countries	SG: 83W<br>P: 85W	SG: 63 (7)<br>P: 63 (8)	SG: 309 (68)<br>P: 315 (67)	SG: 8.2 (0.8)<br>P: 8.1 (0.8)	SG: 12.6 (8.7)<br>P: 12.3 (8.2)
**NCT01042977**		III	24	964	DAPA 10 mg qd	PLB	various	Multicentres in 10 countries	94W	64 (7)	664 (69)	8.1 (0.8)	NA

**NCT01064414**		III	52	272	CANA 100, 300 mg qd	PLB	various	Multicentres in 19 countries	NA	69 (8)	163 (61)	NA	NA
**NCT01081834 CANTATA-M**	Stenlöf et al, 2014	III	52	678	CANA 100, 300 mg qd	PLB->SITA 100 mg qd		90 centres of 17 countries	68W:15A	55 (11)	258 (44)	8.0 (1.0)	4.3 (4.4)
**NCT01087502**		III	52	241	LINA 5 mg qd	PLB->GLM		52 trials in 9 countries	70W	67 (9)	149 (63)	8.1 (0.9)	NA
**NCT01106625 CANTATA-MSU**	Polidori et al, 2014	III	52	469	CANA 100, 300 mg qd	PLB	SU + MET	85 centres in 11 countries	78W:11B	56 (9)	84 (52)	8.2 (1.0)	9.7 (6.1)
**NCT01106651**		III	104	716	CANA 100, 300 mg qd	PLB	various	Multicentres in 17 countries	42US;12Canada:7UK	64 (6)	396 (55)	NA	NA
**NCT01106677 CANTATA-D**	Lavalle-González et al, 2013	III	52	1284	CANA 100, 300 mg qd	PLB/ SITA, SITA 100 mg qd	MET	169 centres in 22 countries	70W:14A	55 (9)	605 (47)	7.9 (0.9)	6.9 (5.3)
**NCT01106690 CANTATA-MP**		III	52	344	CANA 100, 300 mg qd	PLB	MET + PIO	74 centres in 11 countries	74W	57 (10)	216 (63)	7.9 - 8.0 (NA)	10 (NA)
**NCT01107886 SAVOR-TIMI 53**	Mosenzon et al, 2013, NEJM	IV	113	16492	SAXA 5mg qd	PLB	various	788 centres in 26 countries	75W:22H	65 (9)	11043 (67)	8.0 (1.4)	11.9 (8.9)
**NCT01131676 EMPA-REG OUTCOME**	Zinman et al, 2014, 2015	III	140	7028	EMPA 10, 25 mg qd	PLB	various	592 centres in 42 countries	72W:22A	63 (9)	5026 (72)	8.1 (0.8)	<5:5-10:>10(%) 18:25:57
**NCT01137812 CANTATA-D2**	Schernthaner et al, 2013	III	52	756	CANA 300 mg qd	SITA 100 mg qd	SU + MET	140 centres in 17 countries	64W:18A	57 (10)	422 (56)	8.1 (0.9)	9.6 (6.2)
**NCT01164501 EMPA-REG RENAL**	Barnett et al, 2014	III	52	741	EMPA 10, 25 mg qd	PLB	various	127 centres in 15 countries	CKD2: 68W:27A CKD3: 56W:41A	CKD2: 63 (8) CKD3: 65 (9)	CKD2: 177 (61) CKD3: 213 (57)	CKD2: 8.0 (0.8) CKD3: 8.0 (0.7)	≤1:1-5:5-10:>10 CKD2: 1:18:29:52 CKD3: 1:12:20:67
**NCT01189890**	Hartly et al, 2015	III	30	480	SITA 100 mg qd	GLM 1-6 mg qd		Multicenters in 12 countries	D: 61W:25M S: 54W:32M	D: 71 (5) S: 71 (5)	D: 93 (47) S: 77 (40)	D: 7.8 (0.7) S: 7.8 (0.7)	D: 8.0 (5.6) S: 9.4 (7.3)
**NCT01194830**	Thrasher et al, 2012	III	24	226	LINA 5 mg qd	PLB	various	Multicenters in US	90B	54 (10)	121 (54)	8.8 (1.1)	<1:<5:<10:>10(%) 7:37:25:31
**NCT01204294**		III	53	574	LINA 5mg qd	MET	various	Multicenters in Japan		100A	61 (10)	401 (70)	NA
**NCT01214239**		III	24	300	LINA 5 mg qd	PLB		19 centers in 3 countries	87A	54 (10)	175 (59)	8.0 (0.9)	NA
**NCT01233622**		III	24	318	VILD 50 mg bid	PLB	MET + GLM	40 centres in 11 countries	73A:23C	55 (11)	152 (48)	8.8 (0.1)	NA
**NCT01257451 INTERVAL**	Strain et al, 2013	III	24	278	VILD 50 mg bid	PLB	various	45 centres in 7 European countries	D: 97W P: 96W	D: 75 (4) P: 74 (4)	D: 73 (53) P: 53 (38)	D: 7.9 (0.8) P: 7.9 (0.7)	D: 12.2 (7.9) P: 10.6 (6.9)
**NCT01306214 EMPA-REG MDI**	Rosenstock et al, 2014	III	52	566	EMPA 10, 25 mg qd	PLB	INS or MET	Multicentres in 14 countries	94W	57 (10)	256 (45)	8.3 (0.7)	<1:<5:<10:>10(%) <1:9:22:69
**NCT01368081**		III	52	1162	EMPA 10 or 25mg qd	MET up to 2250mg daily	various	Multicentres in Japan	100A	60 (10)	835 (72)	NA	NA
**NCT01541956 VISION**		IV	24	3084	VILDA 50 mg bid	MET up to 1000 mg bid	MET	127 centres in China	100A	57 (11)	1601 (54)	7.2 (0.9)	4.2 (4.2)

**NCT01545388**	III	24	337	MET 500 mg qd or 250 mg bid	PLB	SITA	multicentres in Japan	100A	60 (9)	211 (63)	7.5 (0.8)	NA

Abbreviation

PLB: Placebo; MET: Metfomin; SU: Sulfonylurea; GLM: Glimepiride; GLB: Glibenclamide; GLC: Gliclazide; GLIP: Glipizide; GLY: Glyburide; RSG: Rosiglitazone; PIO: Pioglitazone; SITA: Sitagliptin; VILD: Vildagliptin; SAXA: Saxagliptin; LINA: Linagliptin; DAPA: Dapagliflozin; CANA: Canagliflozin; EMPA: Empagliflozin; P: Placebo; M: Metformin; S: Sulfonyurea; T: Thiazolidinediones; D: Dipeptidyl peptidase 4 inhibitor; SG: Sodium glucose cotransporter 2 inhibitor; W: White; A: Asian; B: Black/ African-American; I: American Indian; H: Hispanic; C: Caucasian; E: Europid
