# Supplementary material for: Comparative effectiveness of oral antidiabetic drugs in preventing cardiovascular mortality and morbidity: A network meta-analysis
Source: PLoS One. 2017 May 25;12(5):e0177646. doi: 10.1371/journal.pone.0177646 (PMC5444626; doi:10.1371/journal.pone.0177646)
Supplement: S3 Table — (PDF) [file pone.0177646.s004.pdf]

S3 Table. Risk of bias assessment

R: Random sequence generation; A: allocation concealment; B1: Blinding of participants and personnel; B2: Blinding of outcome assessment; I: Incomplete outcome data; S: Selective reporting

NCT number	Publication	R	A	B1	B2	I	S
NCT00138619	Rosenstock et al, 2009	U	U	L	L	L	L
NCT00316082		U	U	L	U	U	L
NCT01106625 CANTATA-MSU	Polidori et al, 2014	U	U	L	U	L	L
NCT00740051	NCT00740051	U	U	L	U	U	L
NCT00707993	NCT00707993	U	U	L	L	U	L
NCT00521742	Giles et al, 2010	U	U	L	L	L	L
NCT00138567		U	U	L	U	U	L
NCT01204294		U	H	H	H	U	L
NCT00521820	Giles et al, 2008	U	U	L	L	L	L
NCT01541956 VISION	CLAF237ACN01 Ji et al, 2013	U	H	H	H	L	L
NCT01257451 INTERVAL	Strain et al, 2013	L	L	L	L	L	L
NCT00099866	Schweizer et al, 2007	U	U	L	L	L	L
NCT00509236	Arjona Ferreira et al, 2013, Am J Kidney Dis	L	U	L	L	L	L
NCT01306214 EMPA-REG MDI	Rosenstock et al, 2014	L	L	L	U	U	L
NCT00225264 CHICAGO	Mazzone et al, 2006	U	U	L	U	L	L
NCT00174993 PROactive	Dormandy et al, 2005	L	L	L	H	L	L
NCT00086515	Charbonnel et al, 2006	U	U	L	L	L	L
NCT00313313	Chacra et al, 2010	L	L	L	U	L	L
NCT00500955		L	U	L	U	L	L
NCT00790205 TECOS	Green et al, 2015	L	L	L	L	L	L
NCT00295633	Hollander et al, 2009	L	L	L	U	L	L
NCT00279045 ADOPT	Kahn et al, 2006	L	L	L	L	L	L
NCT00513630 SPREAD-DIMCAD	Hong et al, 2013	L	L	L	U	L	L
NCT00094770	Seck et al, 2010	L	U	L	L	L	L
NCT01006590 PROMPT	Hermans et al, 2012	U	U	L	U	L	L
NCT00286468	Pratley et al, 2009, Diabetes, Obesity and Metabolism	U	U	L	U	L	L

NCT number	Publication	R	A	B1	B2	I	S
NCT01131676 EMPA-REG OUTCOME	Zinman et al, 2014, 2015	L	L	L	L	L	L
NCT00106704	Hermansen et al, 2007	U	U	L	L	L	L
NCT00614939	Nowicki et al, 2011	L	L	L	U	L	L
NCT00602472	Owens et al, 2011	U	U	L	U	L	L
NCT00673231	Wilding et al, 2014	L	U	L	U	L	L
NCT01107886 SAVOR-TIMI 53	Mosenzon et al, 2013, NEJM	L	L	L	L	L	L
NCT01042977		U	U	L	L	U	L
NCT00169832 VICTORY	Bertrand et al, 2010	U	U	L	L	L	L
NCT01214239		U	U	L	U	U	L
NCT00575588	Goke et al, 2011	L	L	L	L	L	L
NCT00601250	NCT00601250	U	U	L	U	U	L
NCT00509262	Arjona Ferreira et al, 2013, Diabetes Care	L	U	L	U	L	L
NCT01106677 CANTATA-D	Lavalle-González et al, 2013	L	U	L	U	L	L
NCT01006603 GENERATION	Schernthaner et al, 2015	L	L	L	L	L	L
NCT00379769 RECORD	Avenell et al, 2011	L	H	H	H	L	L
NCT00855166	Bolinder et al, 2014	L	U	L	L	L	L
NCT00528372	Bailey et al, 2015	L	L	L	L	L	L
NCT00099905	Dejager et al, 2007	U	U	L	L	L	L
NCT00501020		L	L	L	U	L	L
NCT00680745	Strojek et al, 2011	L	U	L	L	L	L
NCT01545388	Protocol No. 136, 2014	U	U	L	U	L	L
NCT00894868		U	U	L	U	U	L
NCT00225277 PERISCOPE	Nissen et al, 2008	L	L	L	L	L	L
NCT00968812 CANTATA-SU	Leiter et al, 2015	L	L	L	U	L	L
NCT00359112	Hamann et al, 2008	U	U	L	U	L	L
NCT00286442	Nauck et al, 2009	U	L	L	U	L	L

NCT00432276	Bosi et al, 2011	U	U	L	U	L	L
NCT00663260	Kohan et al, 2013	U	U	L	L	L	L
NCT00646542	Lukashevich et al, 2011	U	U	L	L	L	L
NCT00968708 EXAMINE	White et al, 2013	U	U	L	L	L	L
NCT01081834 CANTATA-M	Stenlöf et al, 2014	U	U	L	U	L	L
NCT01031680	Cefalu et al, 2015	U	U	L	L	L	L
NCT01011868		U	U	L	U	U	L
NCT00996658		U	U	L	U	U	L
NCT00701090	Arechavaleta et al, 2011	L	L	L	L	L	L
NCT01064414		U	U	L	L	U	L
NCT01106651		U	U	L	U	U	L
NCT01368081		U	U	L	U	U	L
NCT00116831 APPROACH	Gerstein et al, 2010	U	U	L	L	U	L
NCT01087502		U	U	L	U	U	L
NCT00106340		U	U	L	L	L	L
NCT01233622		U	U	L	U	U	L
NCT00333723	Campbell et al, 2004	U	U	L	U	L	L
NCT00102388	Foley et al, 2009	U	U	L	L	L	L
NCT00885352	Fonseca et al, 2013	U	U	L	U	L	L
NCT00856284 ENDURE	Prato et al, 2014	U	U	L	L	U	L
NCT00660907	Nauck et al, 2011	L	L	L	U	L	L
NCT00528879	Bailey et al, 2010, 2013	L	L	L	L	L	L
NCT01137812 CANTATA-D2	Schernthaner et al, 2013	L	L	L	L	L	L
NCT00984867	Jabbour et al, 2014	U	U	L	U	L	L
NCT00765830	NCT00765830	U	U	L	L	U	L
NCT01106690 CANTATA-MP		U	U	L	U	L	L

NCT00449930	Aschner et al, 2010	L	U	L	L	L	L
NCT00286494	Pratley et al, 2009, CMRO	L	L	L	U	L	L
NCT00121667	Rosenstock et al, 2013	U	U	L	U	U	L
NCT01194830	Thrasher et al, 2012	U	U	L	U	U	L
NCT00121641	Rosenstock et al, 2013	U	U	L	U	L	L
NCT00641043	NCT00641043	U	U	L	U	U	L
NCT00622284	Gallwitz et al, 2012	L	L	L	L	L	L
NCT00395343	Vilsboll et al, 2010	L	U	L	L	L	L
NCT00698932	NCT00698932	U	U	L	L	U	L
NCT01164501 EMPA-REG RENAL	Barnett et al, 2014	L	L	L	L	L	L
NCT01189890	Hartly et al, 2015	L	L	L	L	L	L
NCT00350779	Dobs et al, 2013	U	U	L	U	U	L
