# Supplementary material for: Comparative effectiveness of oral antidiabetic drugs in preventing cardiovascular mortality and morbidity: A network meta-analysis
Source: PLoS One. 2017 May 25;12(5):e0177646. doi: 10.1371/journal.pone.0177646 (PMC5444626; doi:10.1371/journal.pone.0177646)
Supplement: S1 Fig — (PDF) [file pone.0177646.s005.pdf]

S1 Fig. Risk of bias assessment (summary graph)

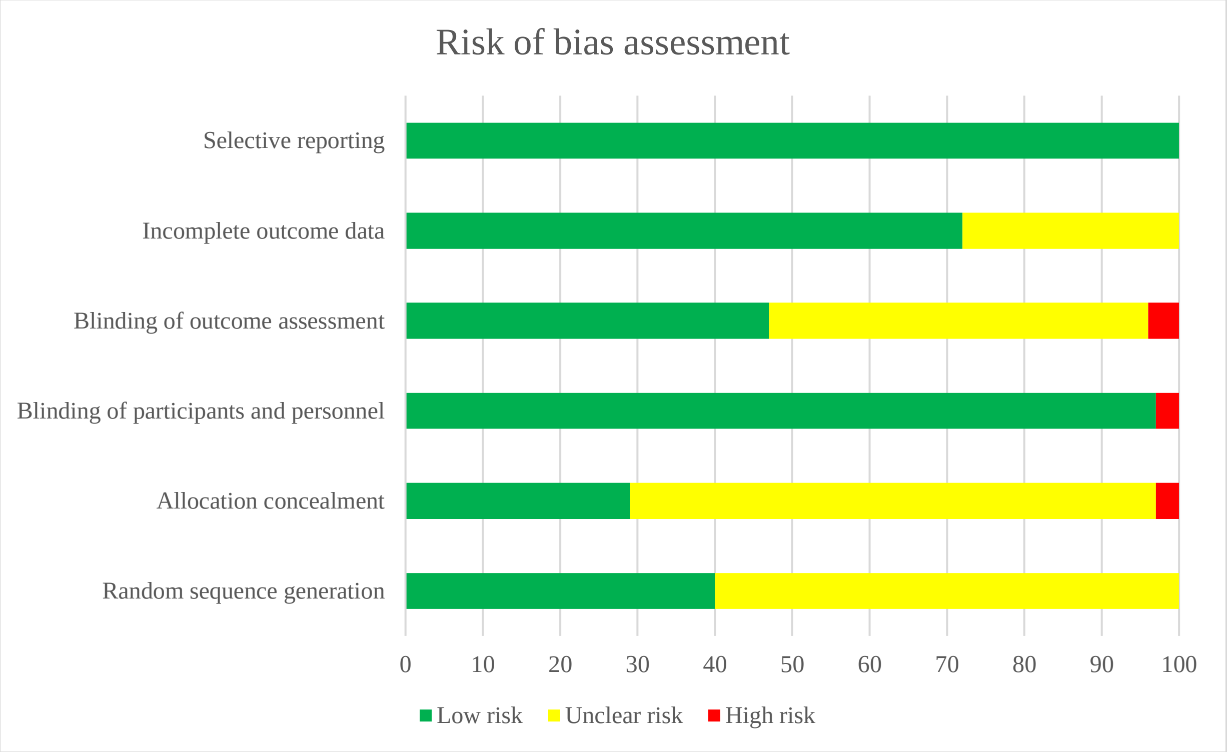

Risk of bias of each included trial was examined by the Cochrane Collaboration's tool. This graph showed the proportion of trials with the judgments for each entry in the tool. Green represents 'low risk of bias'; yellow, 'unclear'; red, 'high risk of bias'.
