# Supplementary material for: Comparative effectiveness of oral antidiabetic drugs in preventing cardiovascular mortality and morbidity: A network meta-analysis
Source: PLoS One. 2017 May 25;12(5):e0177646. doi: 10.1371/journal.pone.0177646 (PMC5444626; doi:10.1371/journal.pone.0177646)
Supplement: S2 Fig — (PDF) [file pone.0177646.s006.pdf]

S2 Fig. Contribution plot for network meta-analysis for all-cause mortality of oral antidiabetic drugs

Direct comparisons in the network (rows: Network meta-analysis estimates)

	AvsB	AvsD	AvsE	AvsF	BvsC	BvsD	BvsE	BvsF	CvsD	CvsE	CvsF	DvsE	EvsF
**Mixed estimates**													
AvsB	0.7	30.3	12.9	1.6	9.4	29.3	4.8	1.3	1.4	7.7	0.3	0.3	
AvsD	0.2	63.7		0.8	1.0	3.2	1.6	0.4	8.1	8.7	0.4	0.8	
AvsE		2.2	92.5		0.2	0.5	0.3		1.5	1.8	0.1	0.2	0.2
AvsF		0.8	1.6	94.0	0.1	0.4	0.1	0.6	0.3	0.4	0.6		1.1
BvsC	0.5	7.8	8.9	0.5	14.6	28.3	4.1	1.1	20.4	13.1	0.5	0.1	
BvsD	0.8	11.4	9.0	1.7	12.6	44.8	5.5	1.5	8.9	3.6	0.1	0.2	
BvsE	0.6	24.9	26.7	1.3	8.1	24.7	4.2	1.1	0.5	7.3	0.2	0.4	0.1
BvsF	0.5	21.3	8.6	30.4	6.7	20.7	3.4	1.1	0.9	5.4	0.4	0.2	0.4
CvsD		18.8	18.0	0.8	6.0	5.9	0.1		31.2	18.2	0.7	0.3	
CvsE	0.1	22.9	24.0	1.0	4.3	2.7	1.2	0.3	20.5	21.7	0.8	0.3	0.1
CvsF	0.1	16.5	14.9	31.6	3.1	1.9	0.7	0.4	14.8	14.5	0.8	0.2	0.4
DvsE	0.1	40.4	40.9	0.4	0.8	2.4	1.2	0.3	6.1	6.6	0.2	0.6	0.1
EvsF		0.8	47.4	48.2	0.1		0.2	0.3	0.6	1.1	0.4	0.1	0.7
**Indirect estimates**													
AvsC	0.2	24.1	22.2	1.2	4.5	3.0	1.1	0.3	21.4	20.9	0.8	0.3	
DvsF	0.1	36.6	5.7	42.4	0.5	2.1	0.9	0.5	4.8	4.9	0.5	0.4	0.5
**Entire network**	0.3	22.1	21.0	16.5	5.1	12.0	2.1	0.7	9.5	9.6	0.5	0.3	0.2
**Included studies**	1	1	19	14	2	3	5	2	8	13	2	2	3

A=placebo. B=metformin. C=sulfonylurea. D=thiazolidinedione (TZD). E=dipeptidyl peptidase-4 (DPP4) inhibitor. F=sodium glucose cotransporter-2 (SGLT2) inhibitor.
