# Supplementary material for: Comparative effectiveness of oral antidiabetic drugs in preventing cardiovascular mortality and morbidity: A network meta-analysis
Source: PLoS One. 2017 May 25;12(5):e0177646. doi: 10.1371/journal.pone.0177646 (PMC5444626; doi:10.1371/journal.pone.0177646)
Supplement: S3 Fig — (PDF) [file pone.0177646.s007.pdf]

S3 Fig. Inconsistency plot for network meta-analysis for all-cause mortality of oral antidiabetic drugs assuming loop-specific heterogeneity estimates.

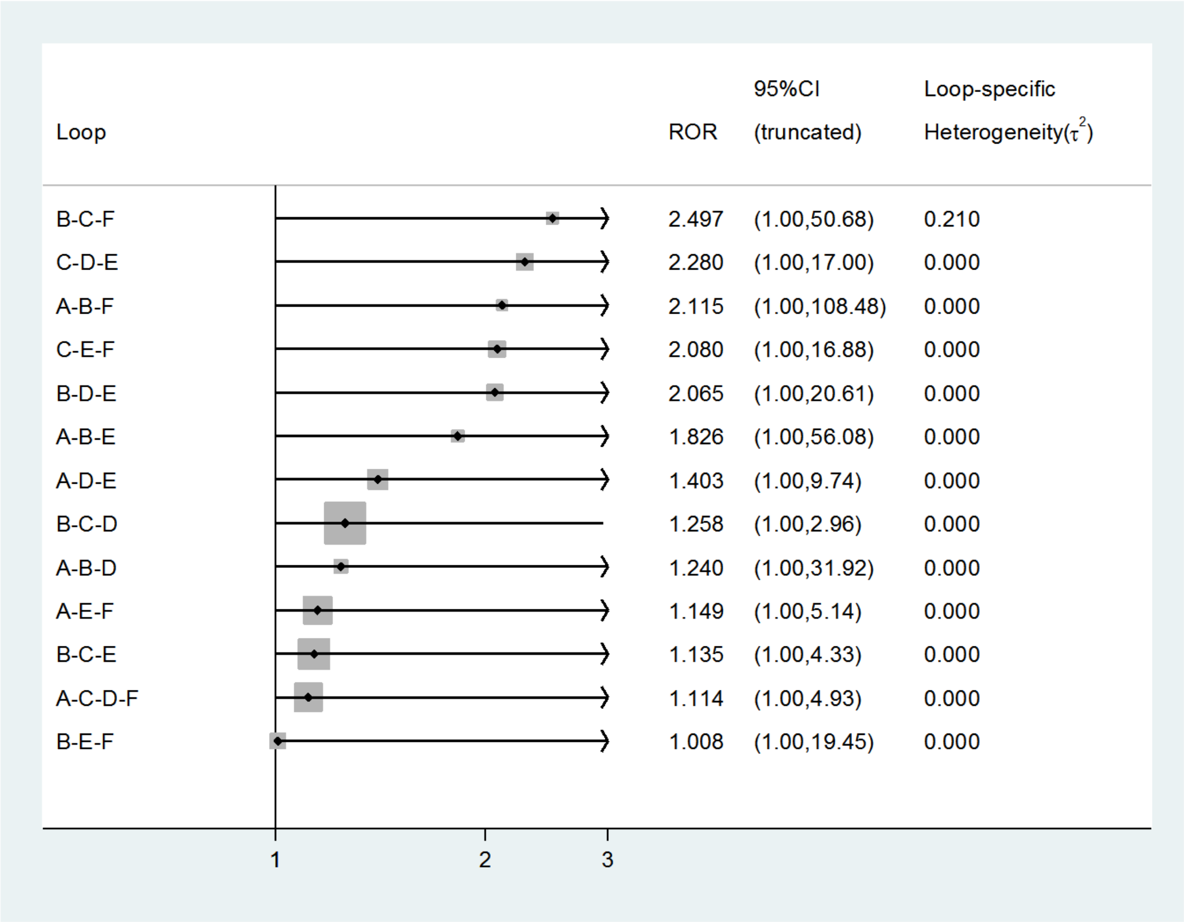

The plot represents that in a total of 13 loops there is none with statistically significant inconsistency as all confidence intervals for RoRs are compatible with zero inconsistency (RoR=1).

A=placebo. B=metformin. C=sulfonylurea. D=thiazolidinedione (TZD). E=dipeptidyl peptidase-4 (DPP4) inhibitor. F=sodium glucose cotransporter-2 (SGLT2) inhibitor.
