# Supplementary material for: Comparative effectiveness of oral antidiabetic drugs in preventing cardiovascular mortality and morbidity: A network meta-analysis
Source: PLoS One. 2017 May 25;12(5):e0177646. doi: 10.1371/journal.pone.0177646 (PMC5444626; doi:10.1371/journal.pone.0177646)

S4 Fig. Comparison-adjusted funnel plot for network meta-analysis for all-cause mortality of oral antidiabetic drugs.

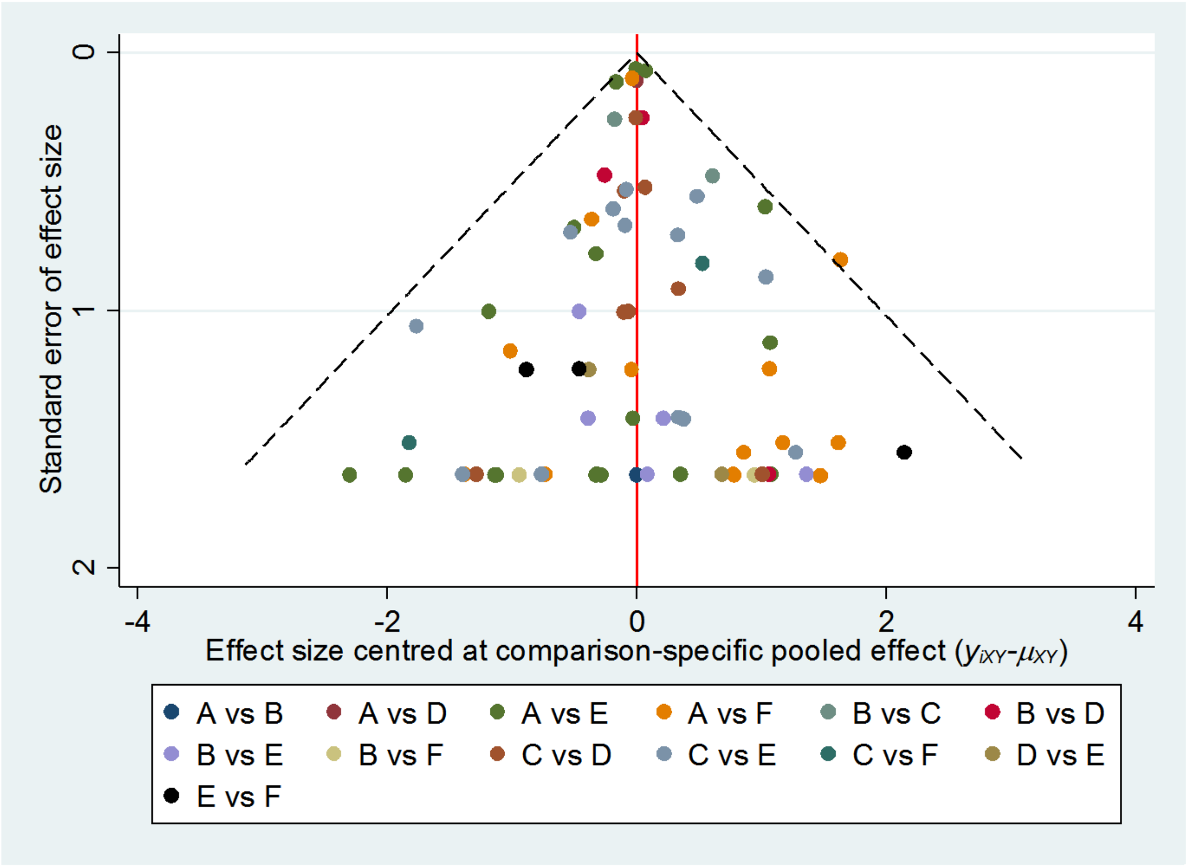

Supplement: S4 Fig — (PDF) [file pone.0177646.s008.pdf]
