# Supplementary material for: Comparative effectiveness of oral antidiabetic drugs in preventing cardiovascular mortality and morbidity: A network meta-analysis
Source: PLoS One. 2017 May 25;12(5):e0177646. doi: 10.1371/journal.pone.0177646 (PMC5444626; doi:10.1371/journal.pone.0177646)

S5 Fig. Network plots and predictive interval plot for sensitivity analysis

Predictive interval plot: The black horizontal lines represent the credible intervals for summary relative risks for each comparison. The vertical line is the line of no difference (relative risk equal to 1). A=PLB=placebo. B=MET=metformin. C=SU=sulfonylurea. D=TZD=thiazolidinedione. E=DPP4=dipeptidyl peptidase-4. F=SGLT2=sodium glucose cotransporter-2.

S5A Fig. Data from double-blinded randomized controlled trials (70 of 73 trials).

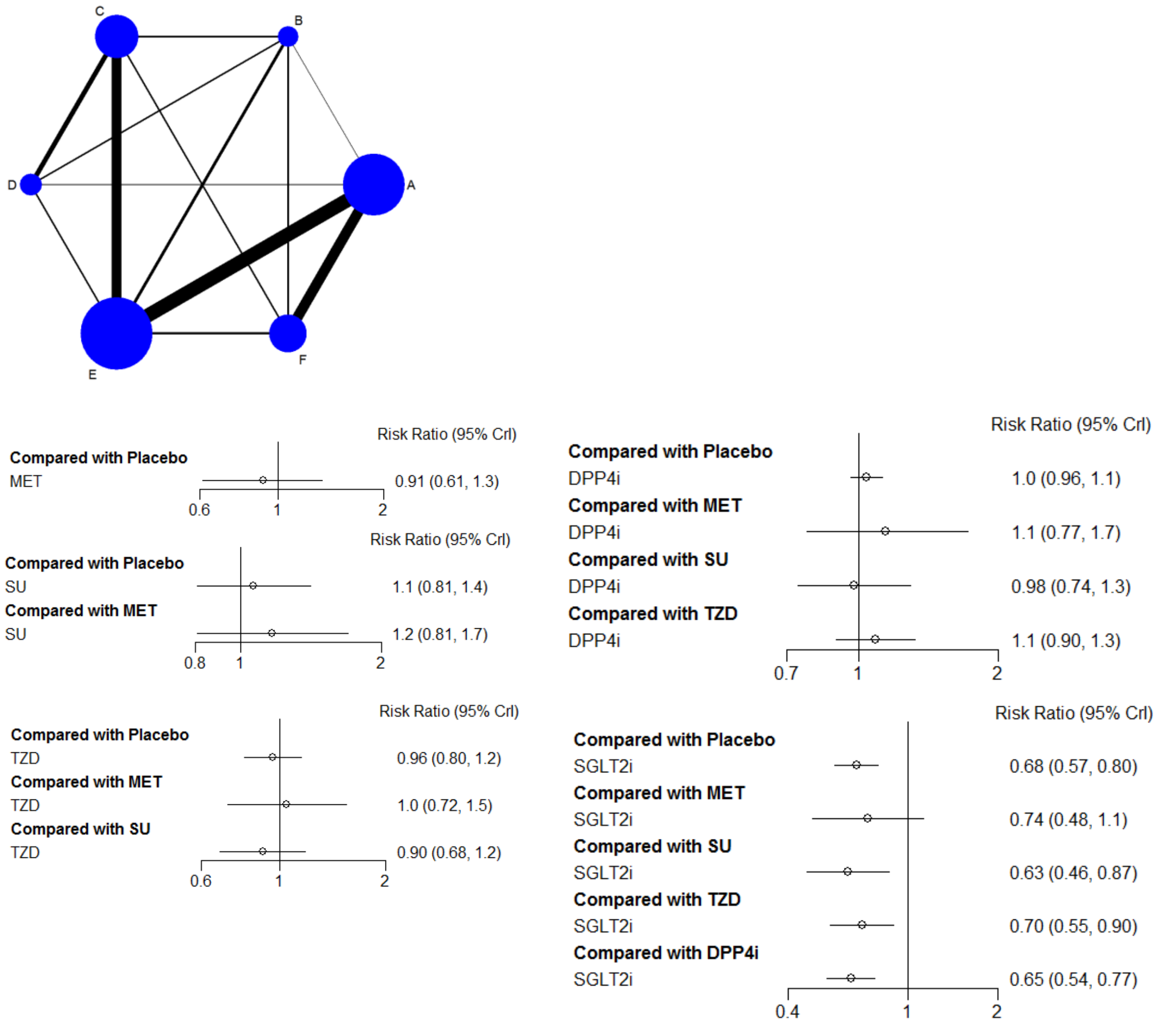

S5B Fig. Data from published articles (52 of 73 trials).

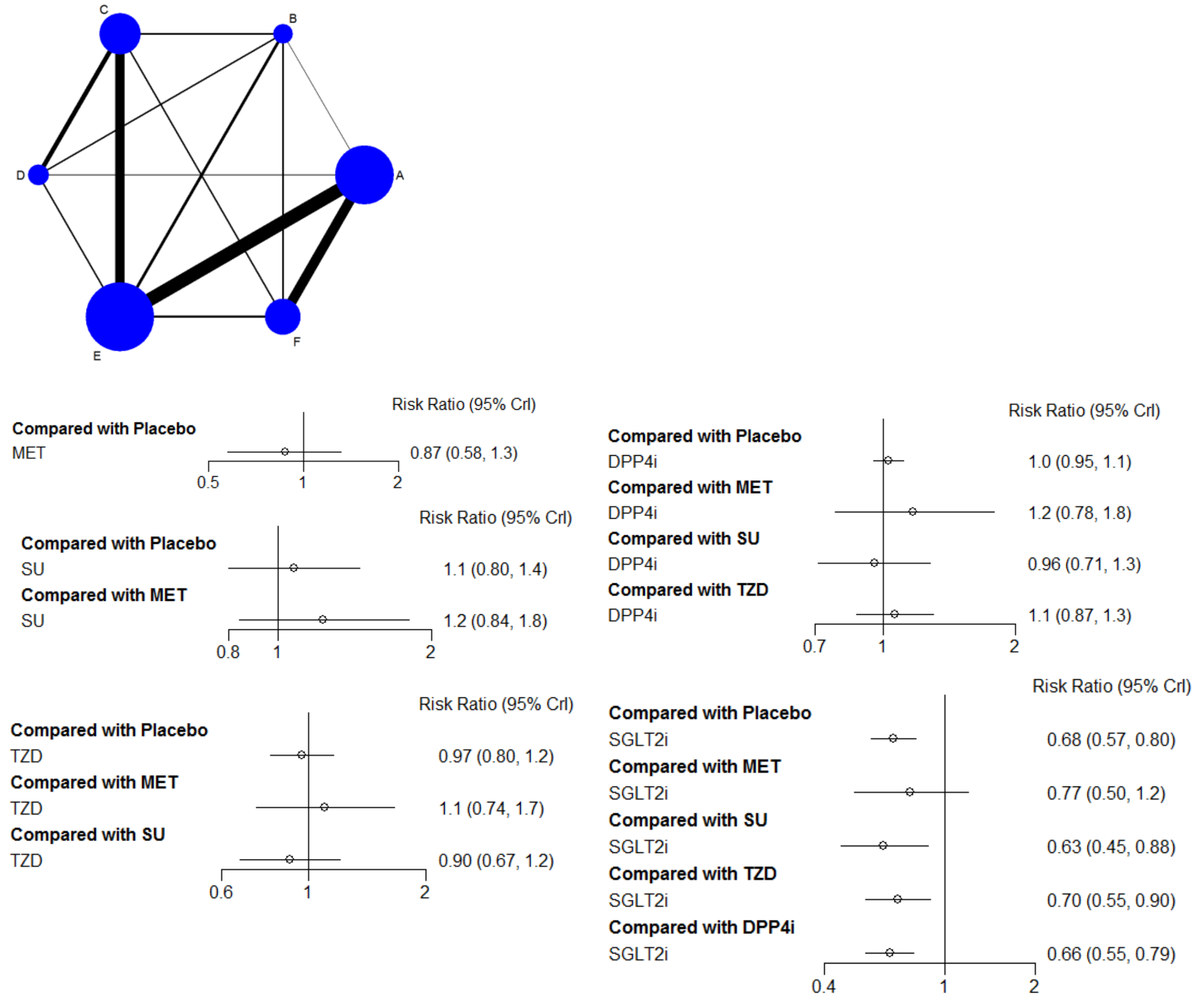

S5C Fig. Data from larger (>500 patients) and longer (>1 year) trials (33 of 73 trials)

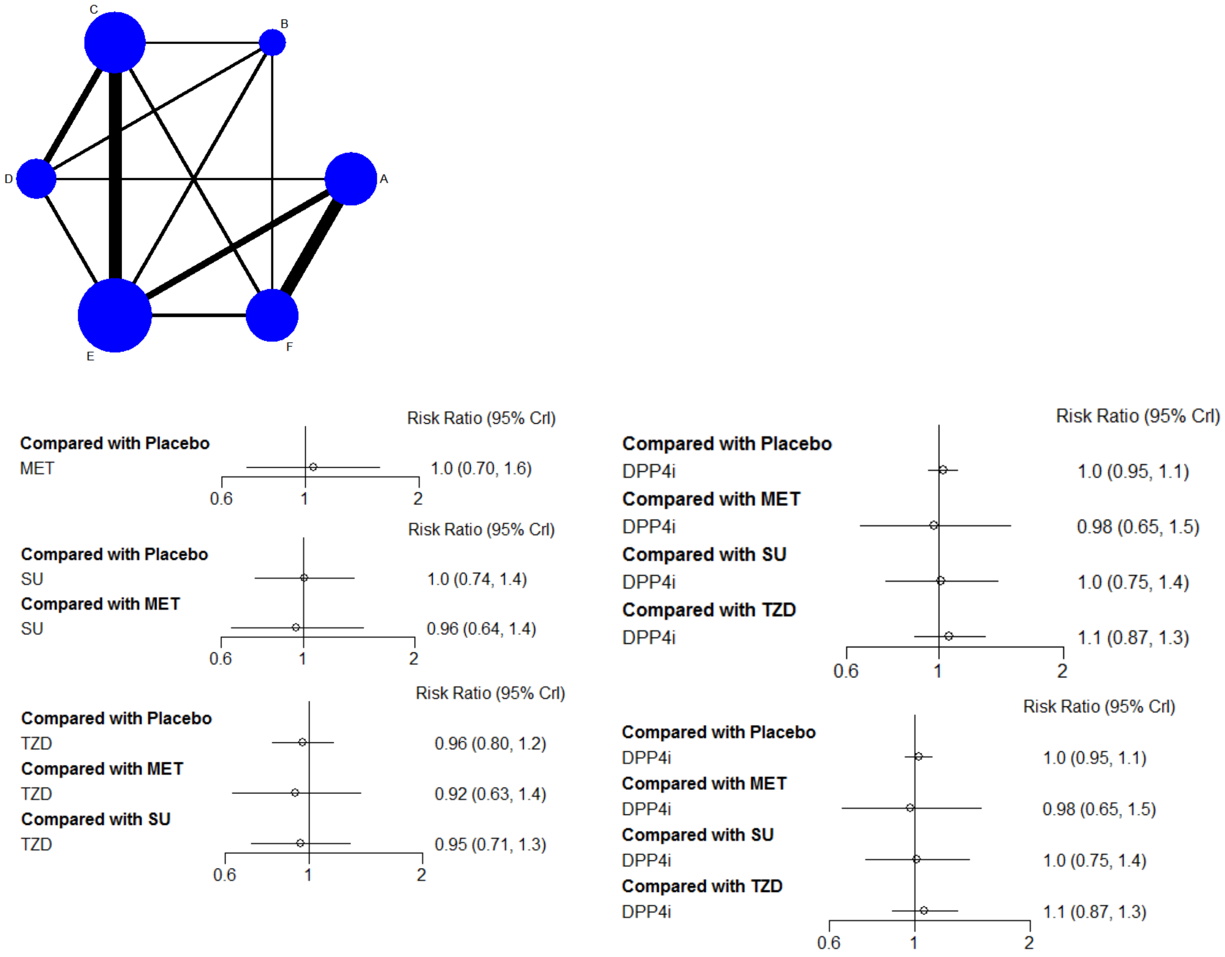

S5D Fig. Data from pre-specified cardiovascular outcome trials (9 of 73 trials)

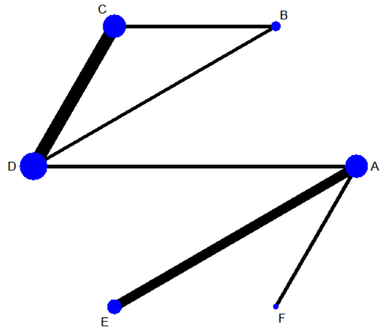

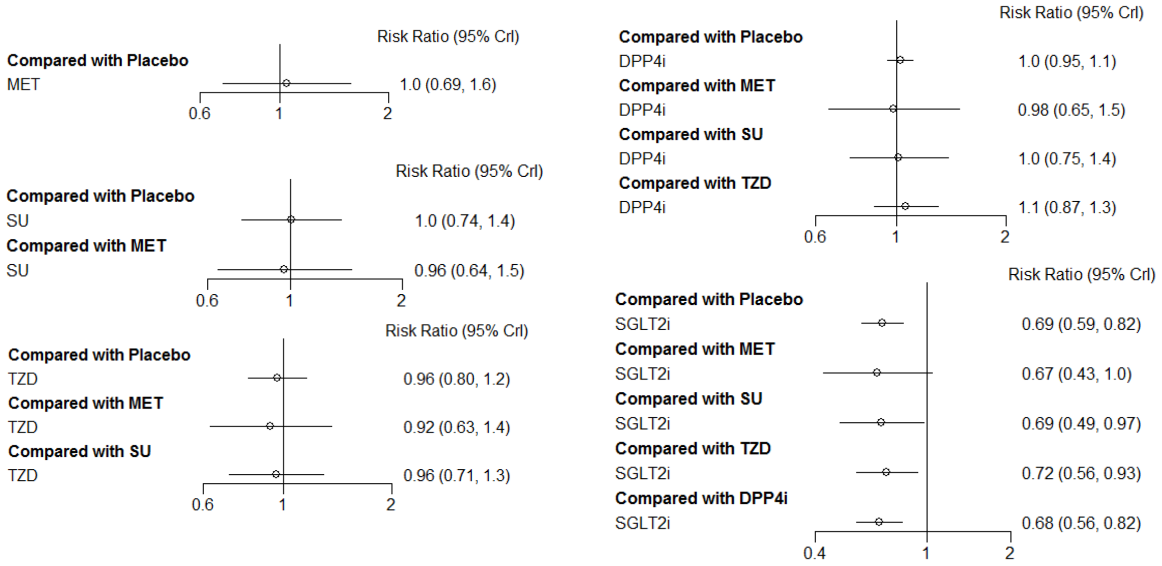

Supplement: S5 Fig — (PDF) [file pone.0177646.s009.pdf]
