# Supplementary material for: Comparative effectiveness of oral antidiabetic drugs in preventing cardiovascular mortality and morbidity: A network meta-analysis
Source: PLoS One. 2017 May 25;12(5):e0177646. doi: 10.1371/journal.pone.0177646 (PMC5444626; doi:10.1371/journal.pone.0177646)

**S6 Fig.** Network plots and predictive interval plot for sub-analysis

Predictive interval plot: The black horizontal lines represent the credible intervals for summary relative risks for each comparison. The vertical line is the line of no difference (relative risk equal to 1). A=PLB=placebo. B=MET=metformin. C=SU=sulfonylurea. D=TZD=thiazolidinedione. E=DPP4=dipeptidyl peptidase-4. F=SGLT2=sodium glucose cotransporter-2.

**S6A Fig.** Younger than 65 years (62 of 73 trials)

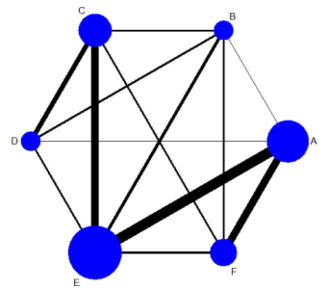


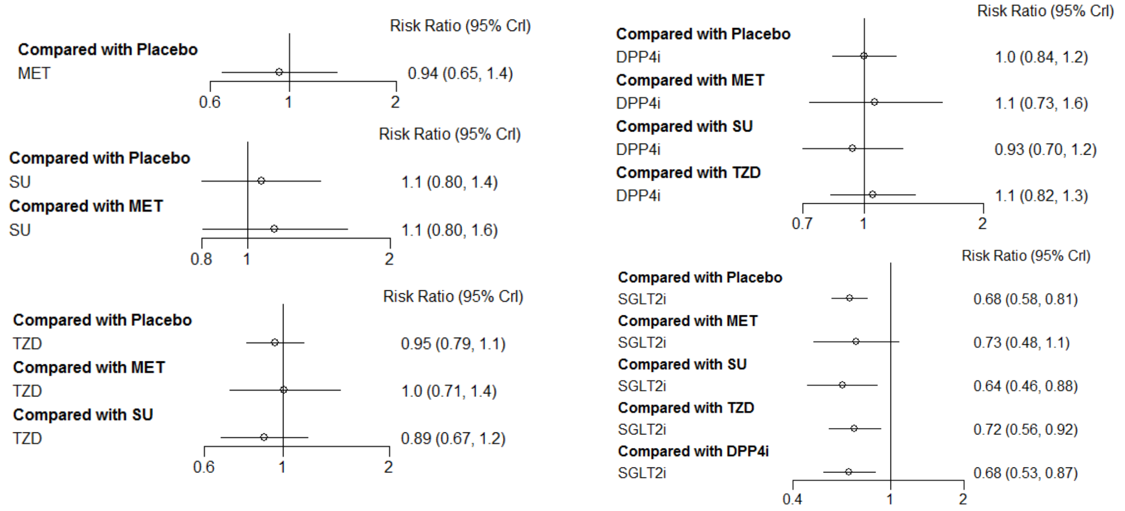


**S6B Fig.** More than 65 years (9 of 73 trials)

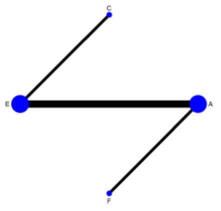

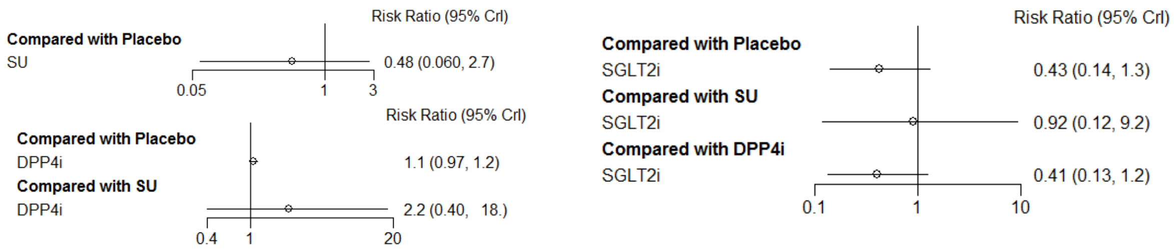

**S6C Fig.** Baseline hemoglobin A1c level below than 8.0 (31 of 73 trials)

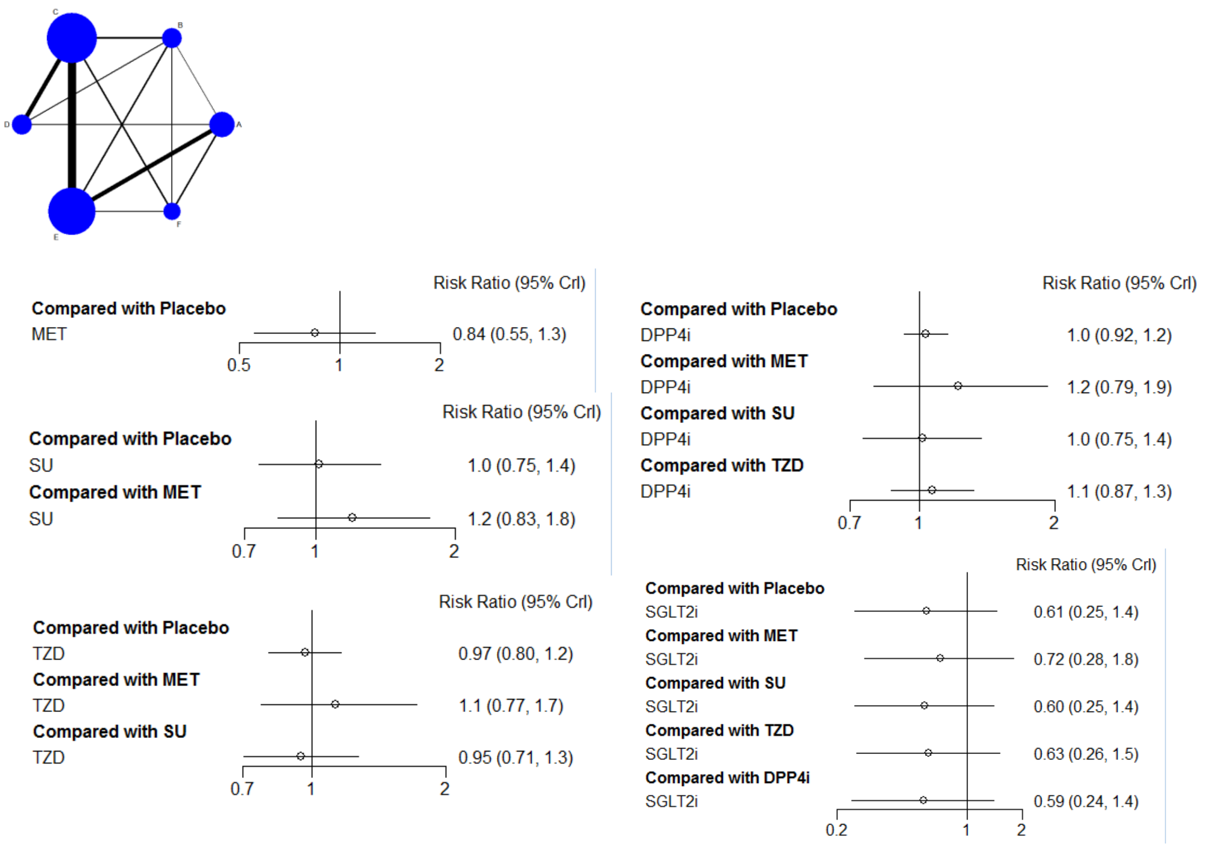


**S6D Fig.** Baseline hemoglobin A1c level more than 8.0 (31 of 73 trials)

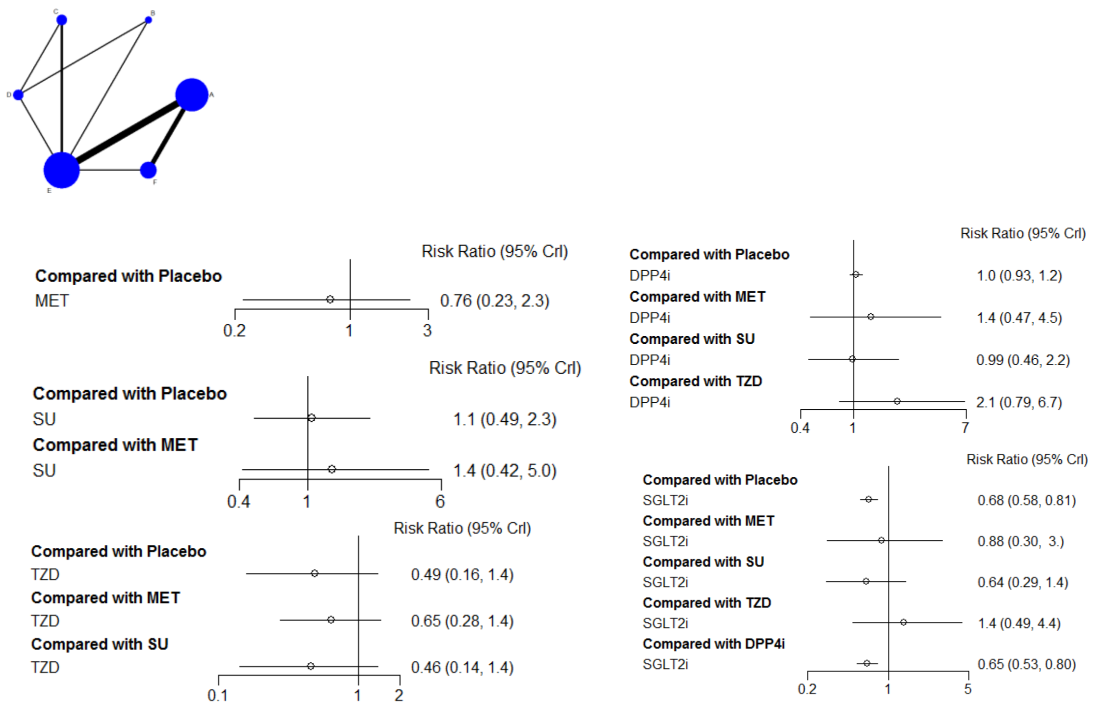

**S6E Fig.** Duration from diabetes diagnosis less than 10 years (43 of 73 trials)

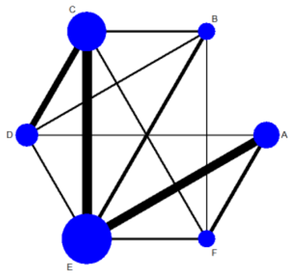


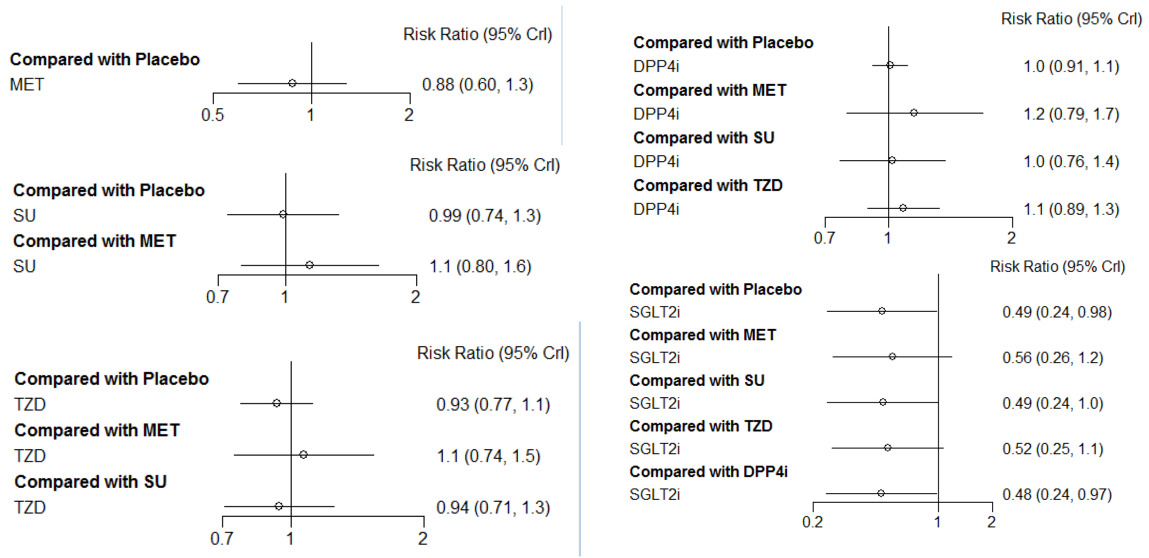


**S6F Fig.** Duration from diabetes diagnosis more than 10 years (14 of 73 trials)

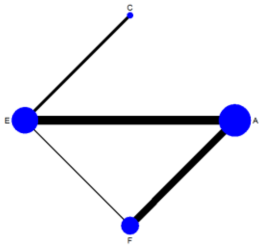


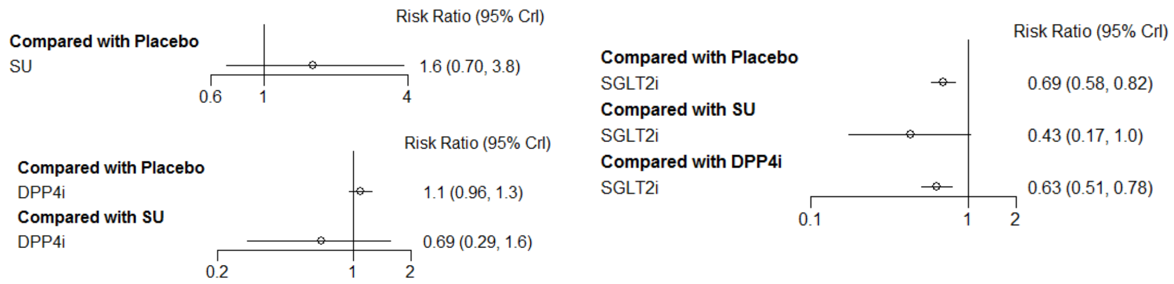

**S6G Fig.** High risk groups (21 of 73 trials)

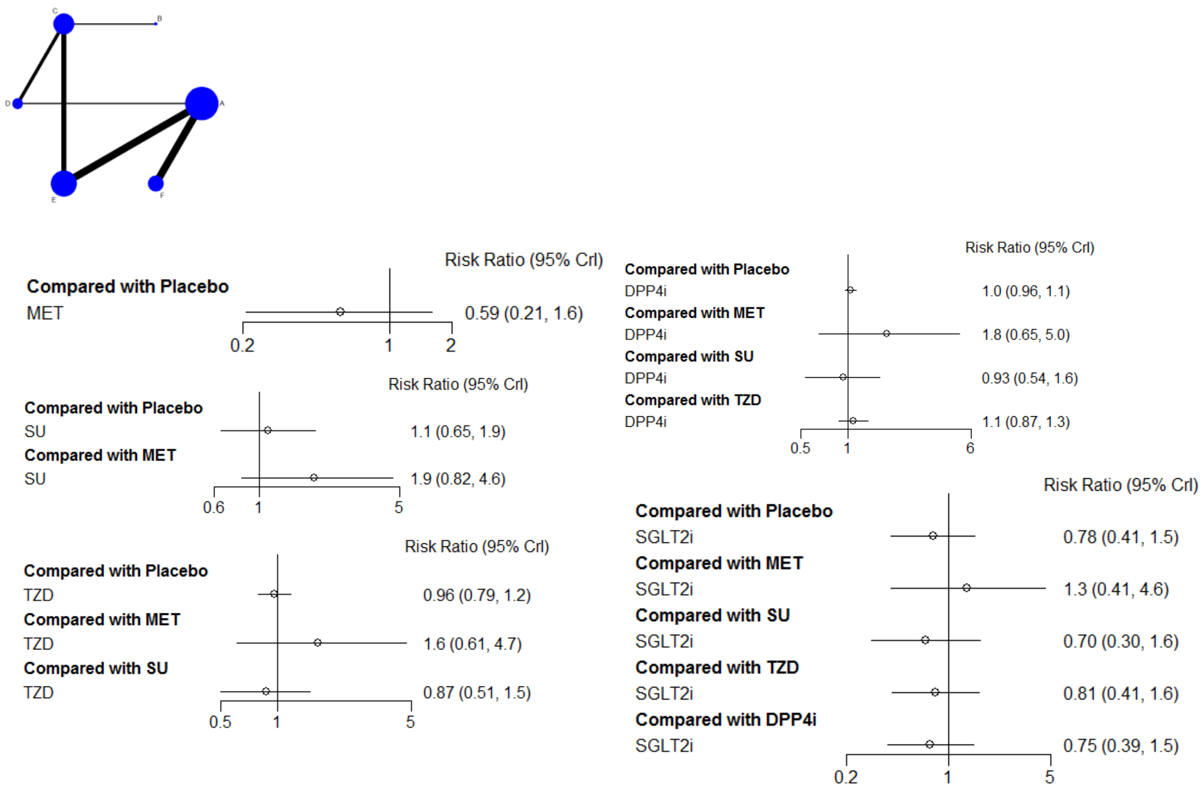


**S6H Fig.** Except rosiglitazone groups (66 of 73 trials)

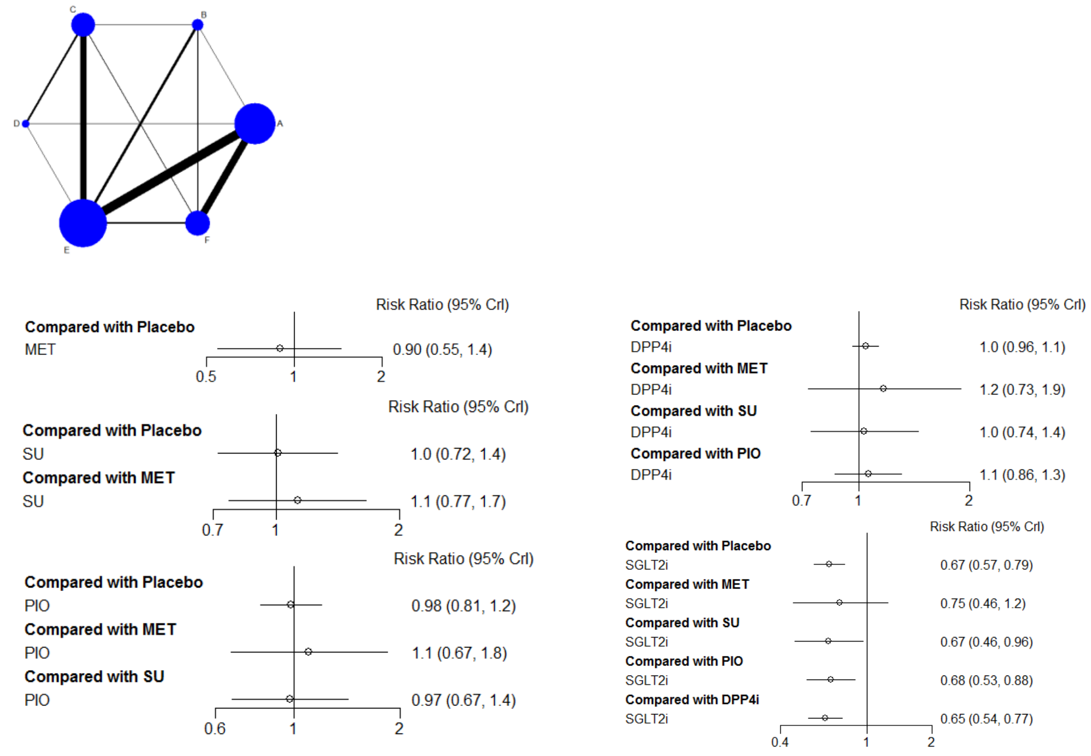

**S6I Fig.** Trial start year after 2008 (39 of 73 trials)

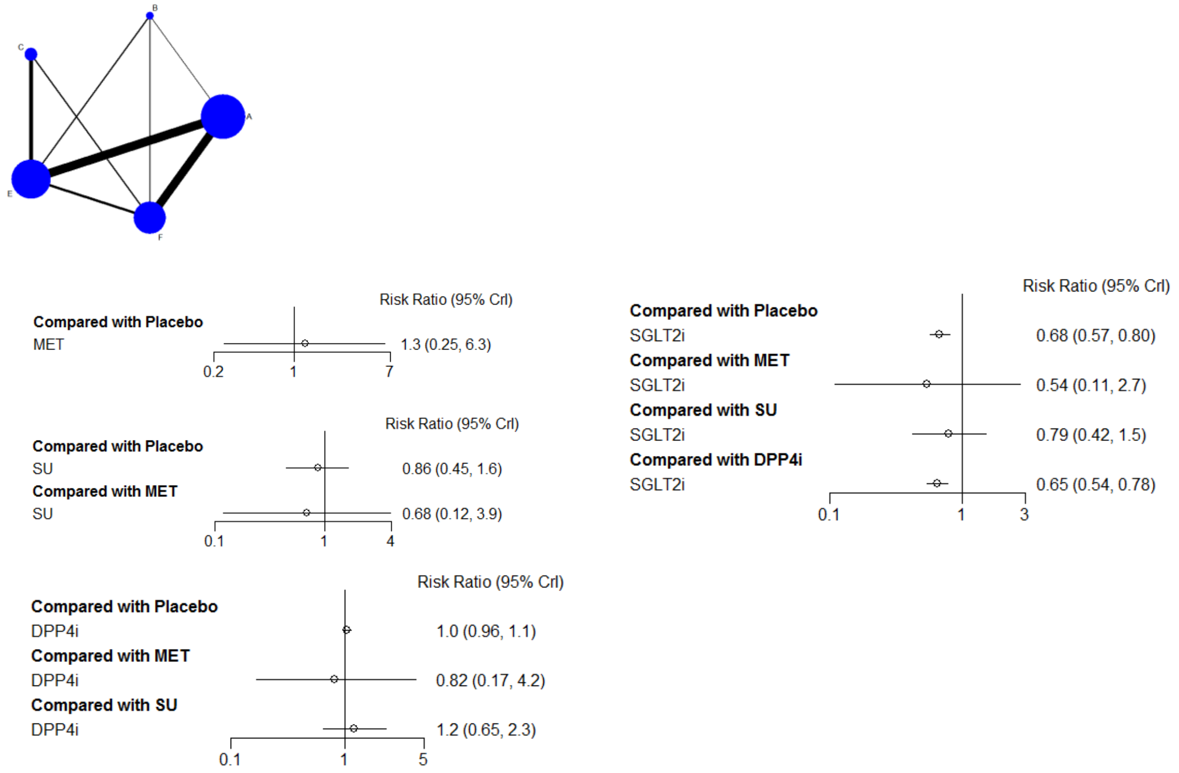


**S6J Fig.** Trial start year before 2008 (33 of 73 trials)

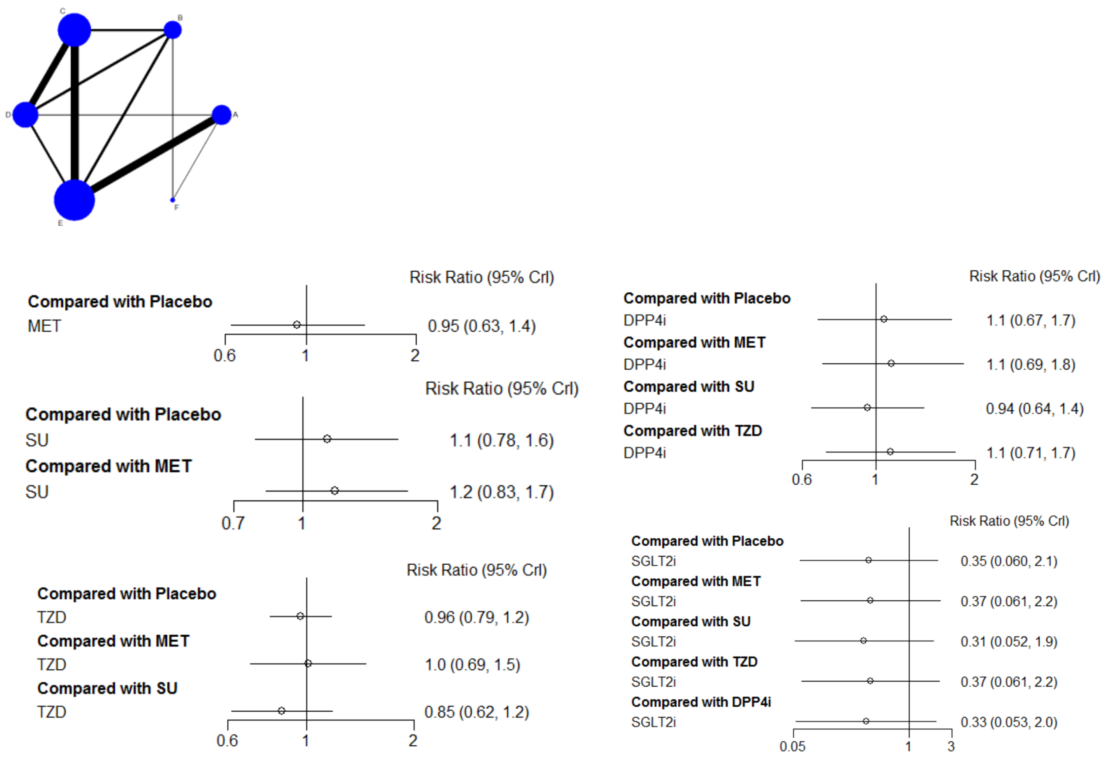

Supplement: S6 Fig — (PDF) [file pone.0177646.s010.pdf]
