# Supplementary material for: Comparative effectiveness of oral antidiabetic drugs in preventing cardiovascular mortality and morbidity: A network meta-analysis
Source: PLoS One. 2017 May 25;12(5):e0177646. doi: 10.1371/journal.pone.0177646 (PMC5444626; doi:10.1371/journal.pone.0177646)

**S7 Fig.** Network meta-analysis for cardiovascular-related mortality

A=placebo. B=metformin. C=sulfonylurea. D=thiazolidinedione (TZD). E=dipeptidyl peptidase-4 (DPP4) inhibitor. F=sodium glucose cotransporter-2 (SGLT2) inhibitor.

**S7A Fig.** Network plot of oral antidiabetic drugs comparison of cardiovascular-related mortality

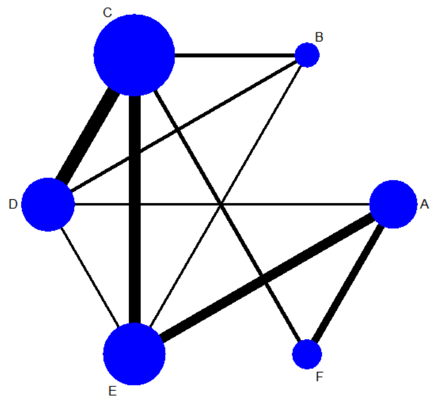


**S7B Fig.** Contribution plot of oral antidiabetic drugs comparison for cardiovascular-related mortality

Direct comparisons in the network

| Network meta-analysis estimates | AvsD | AvsE | AvsF | BvsC | BvsD | BvsE | CvsD | CvsE | CvsF | DvsE |
|---|---|---|---|---|---|---|---|---|---|---|
| Mixed estimates | | | | | | | | | | |
| AvsD | 84.1 | | 0.6 | 0.3 | 0.6 | 0.3 | 4.0 | 3.7 | 0.6 | 0.9 |
| AvsE | 1.6 | 95.2 | | 0.1 | 0.2 | 0.1 | 1.1 | 1.3 | 0.1 | 0.3 |
| AvsF | 0.6 | 0.4 | 97.2 | 0.1 | 0.1 | | 0.5 | 0.3 | 0.9 | |
| BvsC | 3.3 | 2.3 | 1.0 | 58.4 | 13.8 | 3.7 | 10.5 | 6.0 | 1.0 | |
| BvsD | 13.8 | 12.2 | 1.6 | 29.1 | 11.2 | 2.9 | 18.0 | 9.5 | 1.6 | 0.2 |
| BvsE | 20.3 | 21.7 | 1.4 | 22.8 | 8.4 | 2.4 | 12.1 | 9.2 | 1.4 | 0.3 |
| CvsD | 19.9 | 17.1 | 2.7 | 5.2 | 4.2 | 1.0 | 30.4 | 16.4 | 2.7 | 0.3 |
| CvsE | 24.0 | 26.4 | 2.4 | 3.9 | 2.9 | 1.0 | 21.4 | 15.2 | 2.4 | 0.3 |
| CvsF | 19.2 | 12.3 | 31.5 | 3.1 | 2.3 | 0.7 | 17.1 | 11.4 | 2.2 | 0.2 |
| DvsE | 46.1 | 46.4 | 0.3 | 0.2 | 0.5 | 0.2 | 2.8 | 2.7 | 0.3 | 0.6 |
| Indirect estimates | | | | | | | | | | |
| AvsB | 23.1 | 12.6 | 1.6 | 25.3 | 9.4 | 2.6 | 13.9 | 9.7 | 1.6 | 0.2 |
| AvsC | 27.5 | 17.5 | 2.7 | 4.4 | 3.4 | 1.0 | 24.4 | 16.3 | 2.7 | 0.2 |
| BvsF | 17.2 | 9.3 | 26.5 | 19.0 | 7.0 | 1.9 | 10.3 | 7.2 | 1.5 | 0.2 |
| DvsF | 44.5 | 2.4 | 46.9 | 0.2 | 0.4 | 0.2 | 2.3 | 1.8 | 0.8 | 0.5 |
| EvsF | 0.5 | 48.5 | 49.0 | | 0.1 | 0.1 | 0.3 | 0.8 | 0.5 | 0.1 |
| **Entire network** | 22.6 | 20.0 | 16.4 | 12.3 | 4.8 | 1.3 | 12.4 | 8.3 | 1.5 | 0.3 |
| **Included studies** | 1 | 5 | 4 | 2 | 2 | 1 | 7 | 6 | 2 | 1 |

**S7C Fig.** Inconsistency plot for cardiovascular-related mortality of oral antidiabetic drugs assuming loop-specific heterogeneity estimates

The plot represents that in a total of 7 loops there is none with statistically significant inconsistency as all confidence intervals for RoRs are compatible with zero inconsistency (RoR=1).

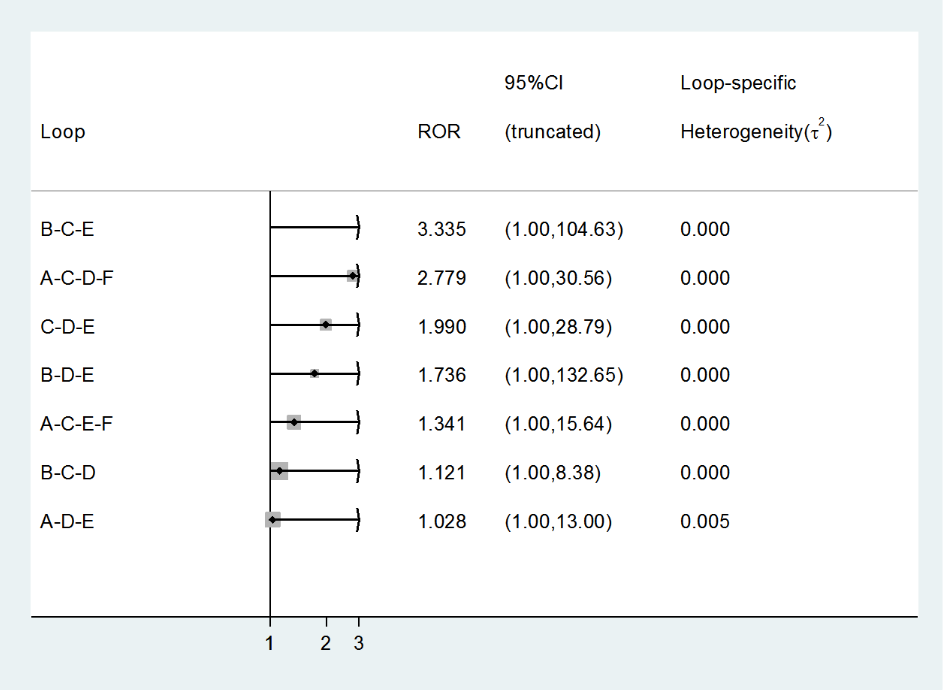


**S7D Fig.** Comparison-adjusted funnel plot for network meta-analysis for cardiovascular-related mortality of oral antidiabetic drugs

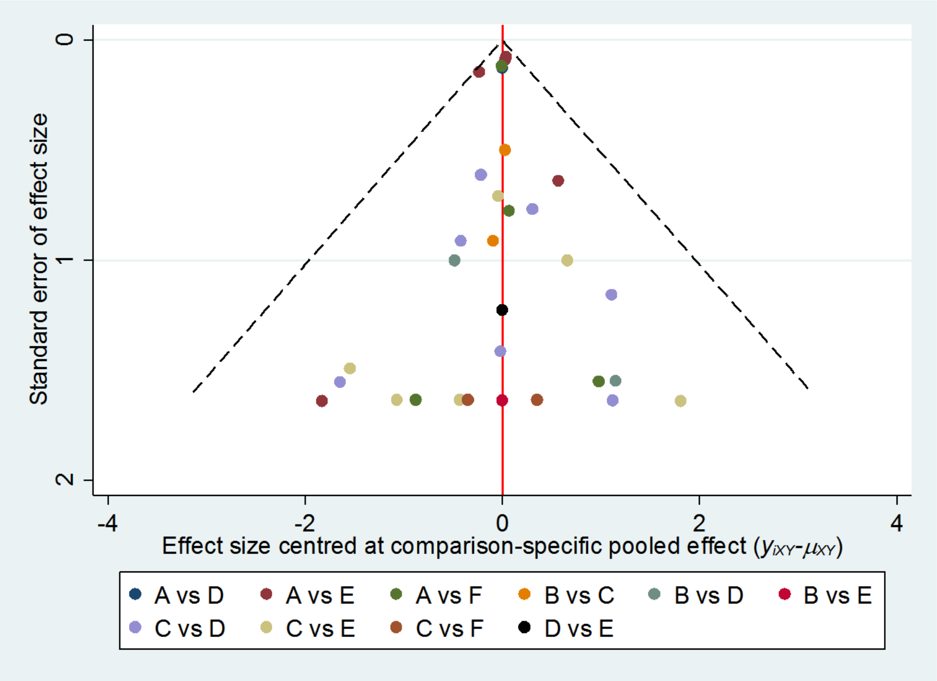

Supplement: S7 Fig — (PDF) [file pone.0177646.s011.pdf]
