# Supplementary material for: Comparative effectiveness of oral antidiabetic drugs in preventing cardiovascular mortality and morbidity: A network meta-analysis
Source: PLoS One. 2017 May 25;12(5):e0177646. doi: 10.1371/journal.pone.0177646 (PMC5444626; doi:10.1371/journal.pone.0177646)

S8 Fig. Network meta-analysis for acute coronary syndrome

A=placebo. B=metformin. C=sulfonylurea. D=thiazolidinedione (TZD). E=dipeptidyl peptidase-4 (DPP4) inhibitor. F=sodium glucose cotransporter-2 (SGLT2) inhibitor.

S8A Fig. Network plot of oral antidiabetic drugs comparison of acute coronary syndrome

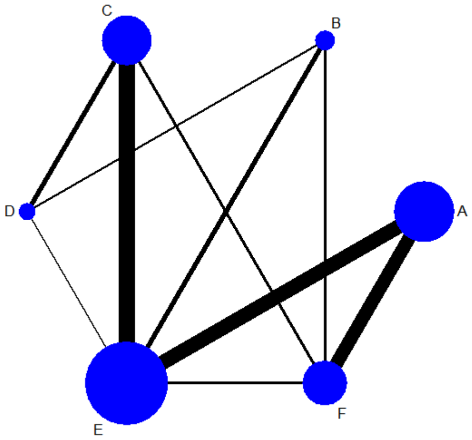

S8B Fig. Contribution plot of oral antidiabetic drugs comparison of acute coronary syndrome

Direct comparisons in the network

Network meta-analysis estimates	AvsE	AvsF	BvsD	BvsE	BvsF	CvsD	CvsE	CvsF	DvsE	EvsF
**Mixed estimates**										
AvsE	52.9	15.7	1.3	2.0	3.3	0.6	11.1	10.6	0.7	1.9
AvsF	12.7	61.9	1.0	1.6	2.6	0.4	9.0	8.6	0.6	1.5
BvsD	0.7	0.7	72.4	6.4	2.5	8.2	6.5	1.7	0.7	0.1
BvsE	4.7	4.7	26.9	9.0	2.9	24.6	22.4	2.1	2.3	0.4
BvsF	14.6	14.6	20.2	5.5	4.8	18.9	9.0	9.9	1.3	1.2
CvsD	0.3	0.3	7.0	5.0	2.0	74.0	7.5	1.7	2.3	
CvsE	7.3	7.3	7.6	6.3	1.3	10.3	50.1	6.5	2.8	0.6
CvsF	21.1	21.1	6.2	1.8	4.4	7.2	19.9	15.7	1.0	1.7
DvsE	5.2	5.2	9.1	7.1	2.0	34.0	30.4	3.6	3.2	0.4
EvsF	30.1	30.1	1.6	2.5	4.2	0.7	14.2	13.5	0.9	2.4
**Indirect estimates**										
AvsB	19.6	10.5	20.3	6.1	3.7	18.9	12.5	6.3	1.5	0.5
AvsC	28.7	14.6	5.7	2.8	2.9	7.0	25.3	10.9	1.3	0.8
AvsD	22.3	11.5	7.3	4.2	3.1	24.5	16.7	7.8	2.0	0.6
BvsC	0.6	0.6	39.5	6.8	2.7	38.6	8.3	2.1	0.9	
DvsF	16.5	16.5	7.7	3.5	4.3	24.3	12.6	11.7	1.7	1.3
**Entire network**	16.3	13.8	15.0	4.8	3.3	19.4	16.9	7.9	1.6	0.9
**Included studies**	12	11	1	4	2	4	13	2	1	2

S8C Fig. Inconsistency plot for acute coronary syndrome of oral antidiabetic drugs assuming loop-specific heterogeneity estimates.

The plot represents that in a total of 6 loops there is none with statistically significant inconsistency as all confidence intervals for RoRs are compatible with zero inconsistency (RoR=1).

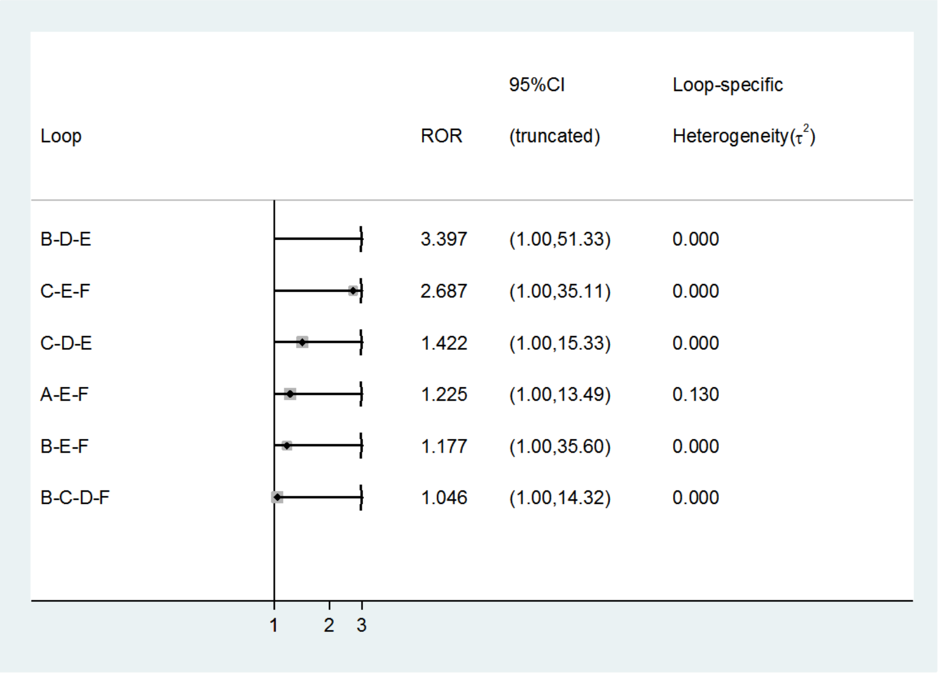

S8D Fig. Comparison-adjusted funnel plot for network meta-analysis for acute coronary syndrome of oral antidiabetic drugs

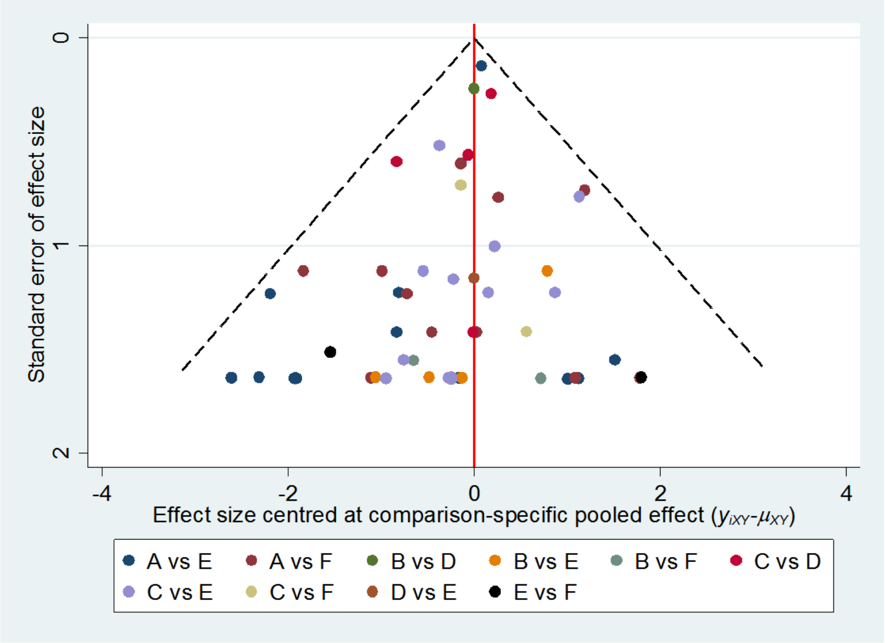

Supplement: S8 Fig — (PDF) [file pone.0177646.s012.pdf]
