# Supplementary material for: Comparative effectiveness of oral antidiabetic drugs in preventing cardiovascular mortality and morbidity: A network meta-analysis
Source: PLoS One. 2017 May 25;12(5):e0177646. doi: 10.1371/journal.pone.0177646 (PMC5444626; doi:10.1371/journal.pone.0177646)

S9 Fig. Network meta-analysis for myocardial infarction

A=placebo. B=metformin. C=sulfonylurea. D=thiazolidinedione (TZD). E=dipeptidyl peptidase-4 (DPP4) inhibitor. F=sodium glucose cotransporter-2 (SGLT2) inhibitor.

S9A Fig. Network plot of oral antidiabetic drugs comparison of myocardial infarction

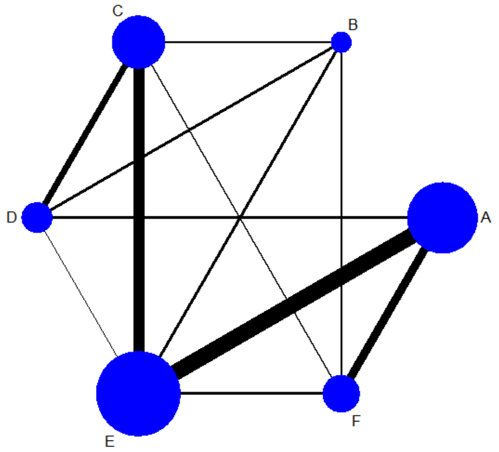

S9B Fig. Contribution plot of oral antidiabetic drugs comparison for myocardial infarction

Direct comparisons in the network

Network meta-analysis estimates	AvsD	AvsE	AvsF	BvsC	BvsD	BvsE	BvsF	CvsD	CvsE	CvsF	DvsE	EvsF
Mixed estimates												
AvsD	51.2	[illegible]	2.3	2.3	3.8	0.9	0.7	11.2	11.9	1.6	0.6	
AvsE	4.2	84.7	[illegible]	0.7	0.9	0.3	0.1	3.1	4.1	0.4	0.2	0.5
AvsF	2.0	2.6	87.3	0.1	0.8	0.1	0.8	1.2	1.2	2.5		1.4
BvsC	7.9	7.6	0.4	18.3	30.6	1.3	0.9	22.6	9.0	1.2	0.1	
BvsD	6.7	5.1	1.7	15.3	52.9	1.7	1.2	11.4	3.4	0.4	0.1	
BvsE	23.1	24.9	1.8	9.5	25.0	1.5	1.0	1.5	10.1	1.0	0.4	0.2
BvsF	20.9	7.8	28.8	8.1	21.9	1.2	1.2	0.7	6.9	1.8	0.2	0.5
CvsD	15.1	13.3	1.8	8.7	8.8	0.1		36.4	13.6	1.8	0.2	
CvsE	21.7	24.2	2.5	4.7	3.6	0.7	0.4	18.5	20.8	2.3	0.4	0.2
CvsF	17.0	13.5	30.5	3.7	2.7	0.4	0.6	14.5	13.5	2.8	0.2	0.6
DvsE	34.5	35.7	1.2	2.0	3.2	0.8	0.4	9.7	10.8	0.9	0.5	0.2
EvsF	1.2	45.0	46.2	0.3		0.2	0.5	1.0	2.9	1.5	0.1	1.0
Indirect estimates												
AvsB	29.5	11.9	2.6	11.0	30.2	1.7	1.2	0.3	10.0	1.3	0.3	
AvsC	24.4	19.8	3.1	5.2	4.1	0.6	0.5	20.5	19.1	2.6	0.3	0.1
DvsF	31.2	7.2	38.4	1.5	2.8	0.5	0.8	7.6	6.9	2.1	0.4	0.6
**Entire network**	20.5	19.6	16.2	6.3	13.1	0.8	0.7	10.3	10.1	1.7	0.3	0.3
**Included studies**	3	21	11	2	3	3	2	8	14	2	1	3

S9C Fig. Inconsistency plot of myocardial infarction on oral antidiabetic drugs assuming loop-specific heterogeneity estimates

The plot represents that in a total of 11 loops there is none with statistically significant inconsistency as all confidence intervals for RoRs are compatible with zero inconsistency (RoR=1).

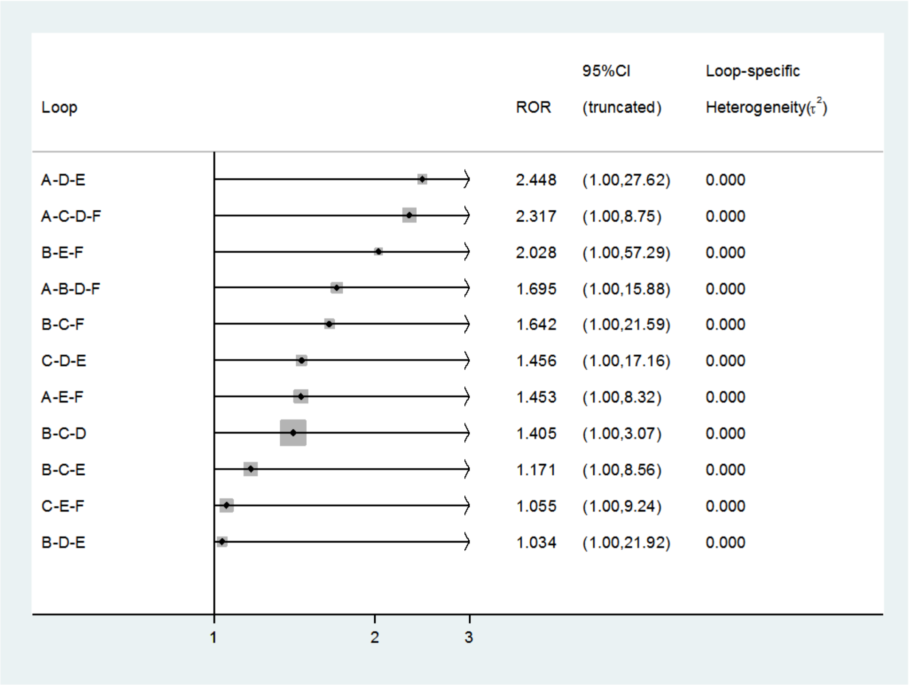

S9D Fig. Comparison-adjusted funnel plot for network meta-analysis for myocardial infarction of oral antidiabetic drugs

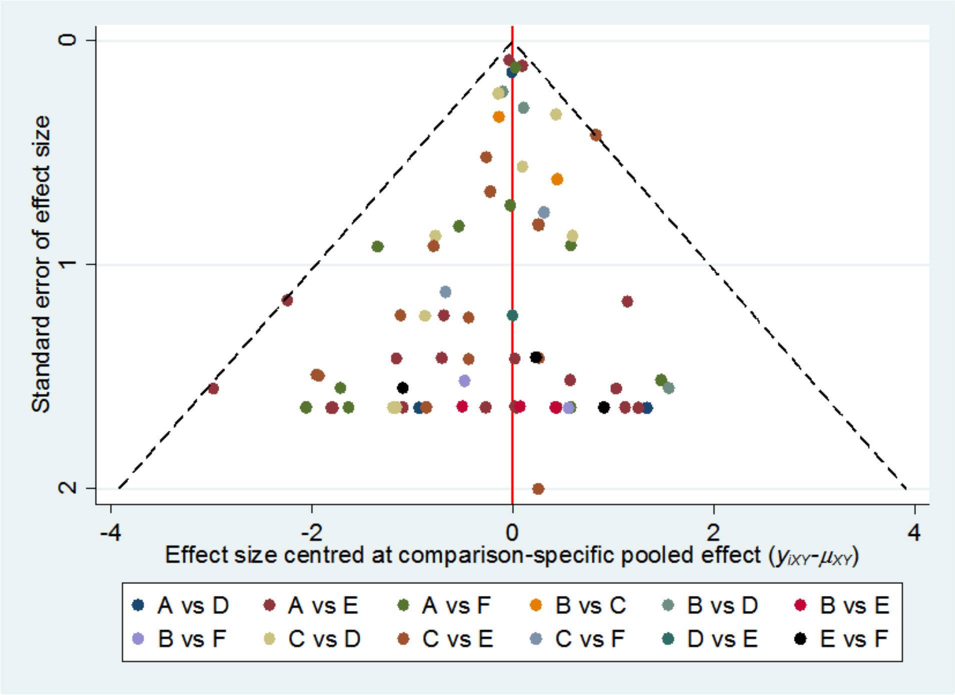

Supplement: S9 Fig — (PDF) [file pone.0177646.s013.pdf]
